# Expression profiling of WD40 family genes including DDB1- and CUL4- associated factor (DCAF) genes in mice and human suggests important regulatory roles in testicular development and spermatogenesis

**DOI:** 10.1186/s12864-020-07016-9

**Published:** 2020-08-31

**Authors:** Bhavesh V. Mistry, Maha Alanazi, Hanae Fitwi, Olfat Al-Harazi, Mohamed Rajab, Abdullah Altorbag, Falah Almohanna, Dilek Colak, Abdullah M. Assiri

**Affiliations:** 1 Department of Comparative Medicine, King Faisal Specialist Hospital & Research Centre, Riyadh, Saudi Arabia; 2 College of Medicine, Alfaisal University, Riyadh, Saudi Arabia; 3 Biostatistics, Epidemiology and Scientific Computing Department, King Faisal Specialist Hospital & Research Centre, Riyadh, Saudi Arabia; 4 Institute for Research and Medical Consultations, Imam Abdulrahman Bin Faisal University, Dammam, Saudi Arabia

**Keywords:** WD40-repeat, DDB1–CUL4-associated factors, Gene expression, RNA sequencing, Testis, Ubiquitination, Spermatogenesis

## Abstract

**Background:**

The WD40-repeat containing proteins, including DDB1–CUL4-associated factors (DCAFs), are abundant and conserved proteins that play important roles in different cellular processes including spermatogenesis. DCAFs are subset of WD40 family proteins that contain WDxR motif and have been proposed to function as substrate receptor for Cullin4-RING-based E3 ubiquitin ligase complexes to recruit diverse proteins for ubiquitination, a vital process in spermatogenesis. Large number of WD40 genes has been identified in different species including mouse and human. However, a systematic expression profiling of WD40 genes in different tissues of mouse and human has not been investigated. We hypothesize that large number of WD40 genes may express highly or specifically in the testis, where their expression is uniquely regulated during testis development and spermatogenesis. Therefore, the objective of this study is to mine and characterize expression patterns of WD40 genes in different tissues of mouse and human with particular emphasis on DCAF genes expressions during mouse testicular development.

**Results:**

Publically available RNA sequencing (RNA seq) data mining identified 347 and 349 WD40 genes in mouse and human, respectively. Hierarchical clustering and heat map analyses of RNA seq datasets revealed differential expression patterns of WD40 genes with around 60–73% of the genes were highly or specifically expressed in testis. Similarly, around 74–83% of DCAF genes were predominantly or specifically expressed in testis. Moreover, WD40 genes showed distinct expression patterns during embryonic and postnatal testis development in mice. Finally, different germ cell populations of testis showed specific patterns of WD40 genes expression. Predicted gene ontology analyses revealed more than 80% of these proteins are implicated in cellular, metabolic, biological regulation and cell localization processes.

**Conclusions:**

We have identified large number of WD40 family genes that are highly or specifically expressed in the testes of mouse and human. Moreover, WD40 genes have distinct expression patterns during embryonic and postnatal development of the testis in mice. Further, different germ cell populations within the testis showed specific patterns of WD40 genes expression. These results provide foundation for further research towards understanding the functional genomics and molecular mechanisms of mammalian testis development and spermatogenesis.

## Background

The WD40-repeat (WDR) containing proteins are characterized by the presence of four or more repeats of 40–60 amino acids stretches that end in tryptophan (W)-aspartate (D) dipeptides [1, 2]. The WDR proteins are most abundant and conserved proteins with critical roles in diverse cellular processes including signal transduction, transcription regulation, DNA damage response, histone modification, cell cycle control, protein degradation and apoptosis [1–7]. The importance of WDR proteins, including DDB1–CUL4-associated factors (DCAFs), is not only demonstrated by their involvement in a diverse array of essential cellular processes, but is also revealed by their association with several human diseases [3, 5, 7–12]. The WD40 repeats fold into β-propeller structure, mostly comprising of seven WD40 repeats, which confer great potential of diverse protein-protein and protein-DNA interactions to form functional multisubunit complexes [5–7]. The genes encoding WDR proteins constitute one of the largest families of genes in the eukaryotic genomes [13–17]. Several systematic genome-wide investigations have been performed to identify and characterize WDR protein encoding genes in human, chimpanzee, mouse, silkworm, plant species and yeast [13, 14, 16–21]. Those investigations have identified 262 non-redundant WDR protein coding genes in human [14], 241 WDR protein coding genes in chimpanzee, 265 WDR protein coding genes in mouse, 172 WDR protein coding genes in silkworm [13], 743 WDR protein coding genes in wheat [18], 237 WDR protein coding genes in Arabidopsis [21], 200 WDR protein coding genes in rice [22] and 83 WDR protein coding genes in yeast [16].

A subgroup of WDR proteins that contain a conserved WDxR motif (also known as DWD or DxR box) within the WD40-repeat is collectively known as DCAFs or DDB1-binding/WD40 domain (DWD) containing proteins or CUL4–DDB1-associated WDR (CDW) proteins [23–25]. The WDxR motifs within the WDR proteins bind to Damaged DNA Binding 1 (DDB1) protein [25]. Most of the DCAF proteins contain two WDXR motifs that are positioned between successive propeller blades and serve as an interaction site for DDB1 protein in the Cullin4-RING-based E3 ubiquitin ligase (CRL4) complexes [24, 25]. However, a few DCAF proteins such as DCAF15, DCAF16, DCAF17, DET1 and DDB1 associated 1 (DDA1) and de-etiolated homolog 1 (DET1) lack the conserved WD40 motif or other known protein interaction domains, but they can still interact with DDB1 [23–26]. The DCAF family proteins have been reported to serve as substrate receptors for the CRL4 complexes to recruit diverse proteins for ubiquitination and thus confer specificity to CRL4s [23, 25, 27, 28]. Protein ubiquitination is a vital reversible post-translational protein modification process that regulates broad range of cellular and developmental processes through proteolytic or non-proteolytic actions [29–34]. In this process, a small highly conserved ~ 8.5 kDa protein (76-amino acid) known as ubiquitin is covalently attached to target proteins by the sequential action of three enzymatic reactions involving ubiquitin-activating enzyme (E1), ubiquitin-conjugating enzyme (E2) and ubiquitin ligase enzyme (E3), in an ATP-dependent manner [32, 35].

Testis is the major male sex organ in which spermatozoa are produced from spermatogonia through a complex and highly regulated molecular and cellular processes, including ubiquitination [33, 34, 36, 37]. Several independent studies have shown the importance of different WDR proteins, including DCAFs, during spermatogenesis [38–46]. In addition, increasing number of studies have shown the significance of ubiquitination in the regulation of normal spermatogenesis [33, 34, 47, 48]. Microarray data mining and expression profiling of ubiquitin E3 ligases (E3s) in the mouse showed expression of 340 putative E3s in the testis including 73 of those E3s were highly or specifically expressed in the testis during different stages of spermatogenesis, suggesting their diverse roles in spermatogenesis [49]. These important findings indicate that WD40 family genes, including DCAF subfamily genes, may have much more specific and complex expression patterns as well as physiological functions during the testicular development and spermatogenesis. Thus, it necessitates for a systematic investigation of WD40 family genes expression analyses across various tissues.

Considering the abundance of WDR proteins and their important roles in many vital cellular processes and disease pathology, we systematically conducted mining and expression profiling of the putative WDR protein encoding genes, including DCAFs, in different tissues of mouse and human with particular emphasis on testicular expression of DCAFs. Mining of mouse and human genome databases and literature search identified 347 and 349 WD40 genes in mouse and human genomes, respectively. Among these WD40 family genes, 60 and 65 belonged to DCAF subfamily genes in the genomes of mouse and human, respectively. RNA seq data analyses detected 289 to 313 WD40 genes with varying transcript levels across different tissues of mouse and human. Interestingly, large number of the WD40 genes was highly or specifically expressed in the testis, suggesting their important functions in the testis. Real time RT-PCR analyses of the mouse DCAF genes for different tissues showed differential expression of these genes with a large proportion of the genes was highly or specifically expressed in the testis. Further, these DCAF genes showed differential expression patterns during different stages of post-natal testis development in the mouse. Together, our data implicate intricate expression patterns for WD40 genes, particularly DCAF genes, reflecting complexity of spermatogenesis and also provide a valuable source to further investigate their functions and potential utilization as infertility biomarkers and/or to develop male contraceptive.

## Results

### Mining of WD40 family genes from the mouse and human genomes

**Fig. 1:**
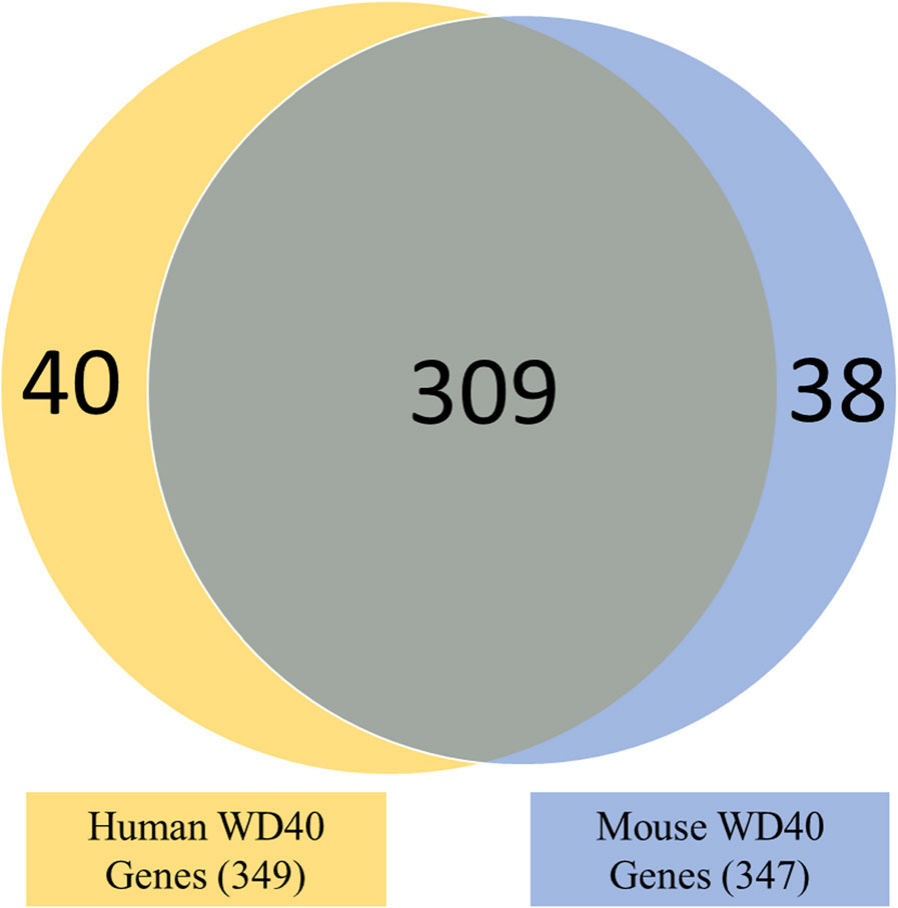
**Venn diagram showing the numbers of unique and common WD40 repeats encoding genes between human and mouse genomes.** Data mining for WD40 repeats encoding genes identified 349 and 347 genes in human and mouse genomes, respectively. The intersection represents the common WD40 genes between the two species

To mine putative WD40 protein encoding genes from the mouse and human genomes, we searched for WD40, DWD, CDW, DCAF, DDB1/2 and CUL4 terms in the publically available databases, genome browsers and scientific publications browser PubMed. Careful selection of WD40 genes resulted in identification of 347 and 349 non-redundant genes in the mouse and the human genomes, respectively (Fig. 1, Supplementary Table S1). Among them, 309 (~ 80%) putative WD40 genes were homologues between mouse and human (Fig. 1, Supplementary Table S1). Such a high proportion of WD40 gene homologues between the two species suggest their conserved roles in different cellular and developmental processes in mouse and human. Among the WD40 family genes, 60 genes (~ 17.3%) in mouse and 65 genes (~ 18.6%) in human genomes belong to DCAF subfamily genes (Table 1). Chromosomal localization analysis of DCAF subfamily genes in mouse genome showed that the genes are broadly distributed on all the chromosomes except chromosomes 3, 17, 18 and Y (Table 1). Similarly, the human genome also showed wide distribution of DCAF genes on all the chromosomes with exception of chromosomes 13, 18, 22 and Y (Table 1). Number of the DCAF genes present on each chromosome varied from 1 to 9 with maximum number of the DCAF genes is present on chromosome 1 in both the species (Table 1). Homology search for the DCAF genes corresponding to mouse and human genes in different species identified 61 DCAF genes in *Bos taurus*, 45 in *Danio rerio*, 60 in *Rattus norvegicus*, 55 in *Xenopus tropicalis*, 5 in *Drosophila melanogaster* and 3 in *Saccharomyces cerevisiae* (Supplementary Table S2).

**Table 1:** The DCAF family genes and their chromosomal location in the human and mouse genomes

Sr. No.	Human DCAF gene symbol	Human gene ID	Human chromosome location	Mouse DCAF gene symbol	Mouse gene ID	Mouse chromosome location
1	*ATG16L1/WDR30*	55,054	2q37.1	*Atg16l1/Wdr30*	77,040	1; 1 D
2	*COP1/RFWD2*	64,326	1q25.1-q25.2	*Cop1/Rfwd2*	26,374	1; 1 H1
3	*CRBN*	51,185	3p26.2	*Crbn*	58,799	6; 6 E1
4	*ERCC8/CSA*	1161	5q12.1	*Ercc8/Csa*	71,991	13; 13 D2.1
5	*DCAF1/VPRBP*	9730	3p21.2	*Dcaf1/Vprbp*	321,006	9; 9 F1
6	*DCAF10/WDR32*	79,269	9p13.2	*Dcaf10/Wdr32*	242,418	4; 4 B1
7	*DCAF11/WDR23*	80,344	14q12	*Dcaf11/Wdr23*	28,199	14 C3; 14 28.19 cM
8	*DCAF12/WDR40A*	25,853	9p13.3	*Dcaf12/Wdr40a*	68,970	4; 4 A5
9	*DCAF12L1*	139,170	Xq25	*Dcaf12l1*	245,404	X; X A4
10	*DCAF12L2*	340,578	Xq25	*Dcaf12l2*	245,403	X; X A4
11	*DCAF13/WDSOF1*	25,879	8q22.3	*Dcaf13/Wdsof1*	223,499	15; 15 B3.1
12	*DCAF14/PHIP*	55,023	6q14.1	*Dcaf14/Phip*	83,946	9; 9 E2
13	*DCAF15*	90,379	19p13.12	*Dcaf15*	212,123	8; 8 C2
14	*DCAF17*	80,067	2q31.1	*Dcaf17*	75,763	2; 2 C2
15	*DCAF19/BRWD1*	54,014	21q22.2	*Dcaf19/Brwd1*	93,871	16; 16 C4
16	*DCAF2/CDT2/DTL*	51,514	1q32.3	*Dcaf2/Cdt2/Dtl*	76,843	1; 1 H6
17	*DCAF3/AMBRA1*	55,626	11p11.2	*Dcaf3/Ambra1*	228,361	2; 2 E1
18	*DCAF4/WD21A*	26,094	14q24.2	*Dcaf4/Wd21a*	73,828	12; 12 D1
19	*DCAF5/WDR22*	8816	14q24.1	*Dcaf5/Wdr22*	320,808	12; 12 C3
20	*DCAF6/IQWD1*	55,827	1q24.2	*Dcaf6/Iqwd1*	74,106	1; 1 H2.2
21	*DCAF7/WDR68*	10,238	17q23.3	*Dcaf7/Wdr68*	71,833	11; 11 E1
22	*DCAF8/WDR42A*	50,717	1q23.2	*Dcaf8/Wdr42a*	98,193	1 H3; 1 79.54 cM
23	*DCAF9/WDTC1*	23,038	1p36.11	*Dcaf9/Wdtc1*	230,796	4; 4 D2.3
24	*DDA1*	79,016	19p13.11	*Dda1*	66,498	8; 8 B3.3
25	*DDB2*	1643	11p11.2	*Ddb2*	107,986	2; 2 E1
26	*DET1*	55,070	15q26.1	*Det1*	76,375	7; 7 D2
27	*EED/ESC1*	8726	11q14.2	*Eed/Esc1*	13,626	7 D3; 7
28	*FBXW5*	54,461	9q34.3	*Fbxw5*	30,839	2; 2 A3
29	*FBXW8*	26,259	12q24.22	*Fbxw8*	231,672	5; 5 F
30	*GRWD1*	83,743	19q13.33	*Grwd1*	101,612	7; 7 B3
31	*Gβ2/GNB2*	2783	7q22.1	*Gβ2/Gnb2*	14,693	5 G2; 5 76.54 cM
32	*IFRG15/TOR1AIP2*	163,590	1q25.2	*Ifrg15/Tor1aip2*	240,832	1; 1 G3
33	*KATNB1*	10,300	16q21	*Katnb1*	74,187	8; 8 C5
34	*mβTrCP/BTRC*	8945	10q24.32	*Mβtrcp/Btrc*	12,234	19; 19 C3
35	*NLE1*	54,475	17q12	*Nle1*	217,011	11; 11 C
36	*NUP43*	348,995	6q25.1	*Nup43*	69,912	10; 10 A1
37	*PAFAH1B1/LIS1*	5048	17p13.3	*Pafah1b1/Lis1*	18,472	11 B5; 11 45.76 cM
38	*PWP1*	11,137	12q23.3	*Pwp1*	103,136	10; 10 C1
39	*RBBP4*	5928	1p35.1	*Rbbp4*	19,646	4; 4 D2.2
40	*RBBP5*	5929	1q32.1	*Rbbp5*	213,464	1; 1 E4
41	*RBBP7*	5931	Xp22.2	*Rbbp7*	245,688	X; X F4
42	*SMU1*	55,234	9p21.1	*Smu1*	74,255	4; 4 A5
43	*TLE1*	7088	9q21.32	*Tle1*	21,885	4; 4 C3
44	*TLE2*	7089	19p13.3	*Tle2*	21,886	10; 10 C1
45	*TLE3*	7090	15q23	*Tle3*	21,887	9; 9 B
46	*TRPC4AP*	26,133	20q11.22	*Trpc4ap*	56,407	2 H1; 2 77.26 cM
47	*WDR12*	55,759	2q33.2	*Wdr12*	57,750	1; 1 C2
48	*WDR26*	80,232	1q42.11-q42.12	*Wdr26*	226,757	1; 1 H4
49	*WDR39/CIAO1*	9391	2q11.2	*Wdr39/Ciao1*	26,371	2 F1; 2 61.86 cM
50	*WDR5*	11,091	9q34.2	*Wdr5*	140,858	2; 2 A3
51	*WDR51B/POC1B*	282,809	12q21.33	*Wdr51b/Poc1b*	382,406	10; 10 C3-D1
52	*WDR53*	348,793	3q29	*Wdr53*	68,980	16; 16 B2
53	*WDR57/SNRNP40*	9410	1p35.2	*Wdr57/Snrnp40*	66,585	4; 4 D2.2
54	*WDR59*	79,726	16q23.1	*Wdr59*	319,481	8; 8 E1
55	*WDR5B*	54,554	3q21.1	*Wdr5b*	69,544	16; 16 B3
56	*WDR61*	80,349	15q25.1	*Wdr61*	66,317	9; 9 A5.3
57	*WDR76*	79,968	15q15.3	*Wdr76*	241,627	2; 2 E5
58	*WDR82*	80,335	3p21.2	*Wdr82*	77,305	9; 9 F1
59	*WSB1*	26,118	17q11.1	*Wsb1*	78,889	11; 11 B5
60	*WSB2*	55,884	12q24.23	*Wsb2*	59,043	5; 5 F
61	*DCAF16*	54,876	4p15.31	Absent in mouse	–	–
62	*DCAF4L1*	285,429	4p13	Absent in mouse	–	–
63	*DCAF4L2*	138,009	8q21.3	Absent in mouse	–	–
64	*DCAF8L1*	139,425	Xp21.3	Absent in mouse	–	–
65	*DCAF8L2*	347,442	Xp21.3	Absent in mouse	–	–

### RNA sequencing data analyses of WD40 family genes identified testis-enriched genes in mouse and human

**Fig. 2:**
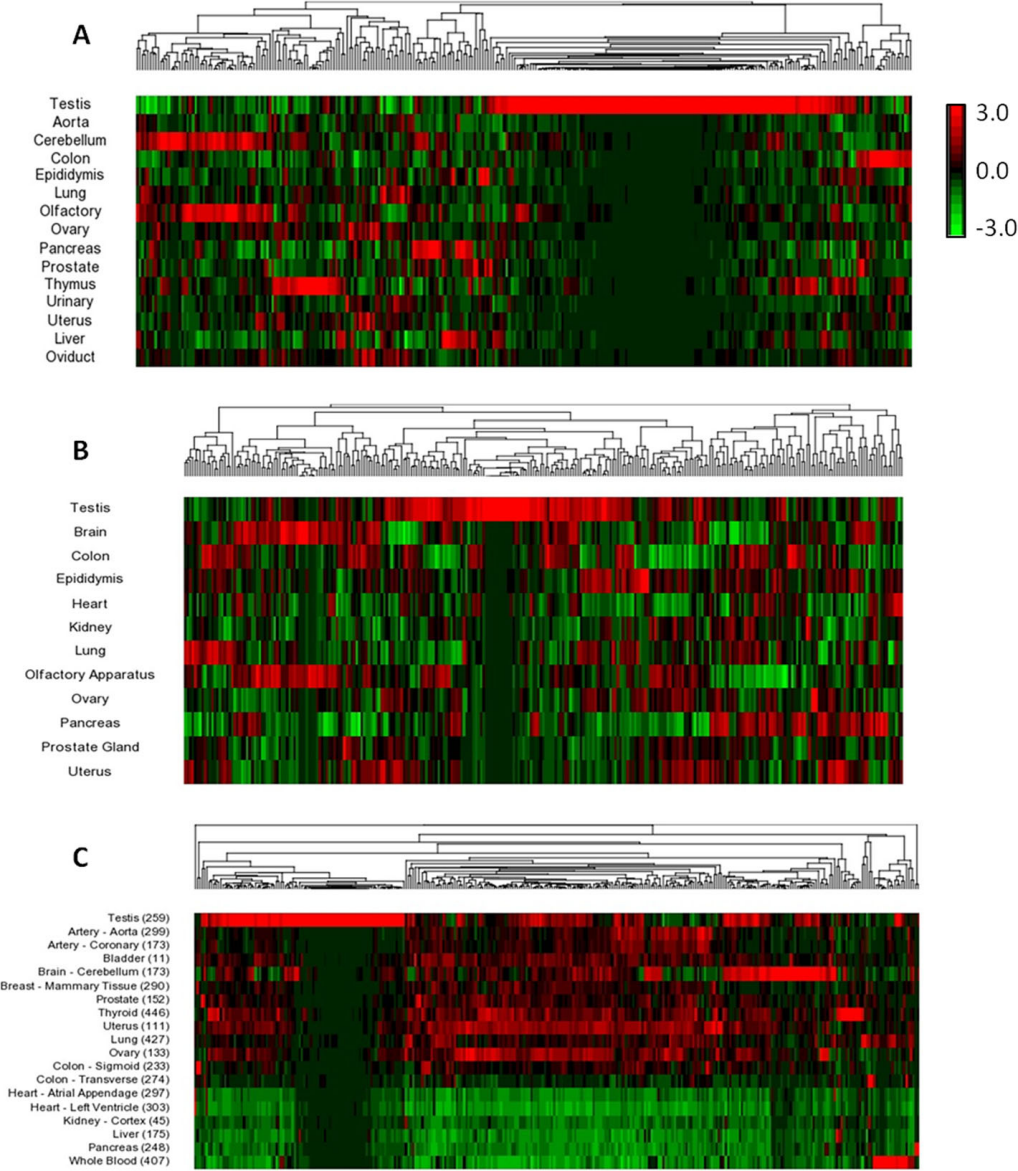
Unsupervised hierarchical clustering of mRNA expression profile of the WD40 repeat encoding genes family across different tissues of mouse and human. Unsupervised hierarchical clustering analyses of mouse **a** and human **b**, **c** WD40 genes using FANTOM5 RNA seq datasets **a**, **b** and GTEx RNA seq dataset **c** showed differential mRNA expression patterns in different tissues of adult mouse and human. Samples are denoted in rows and genes are denoted in columns. Red and green denote highly and weakly expressed genes, respectively

**Fig. 3:**
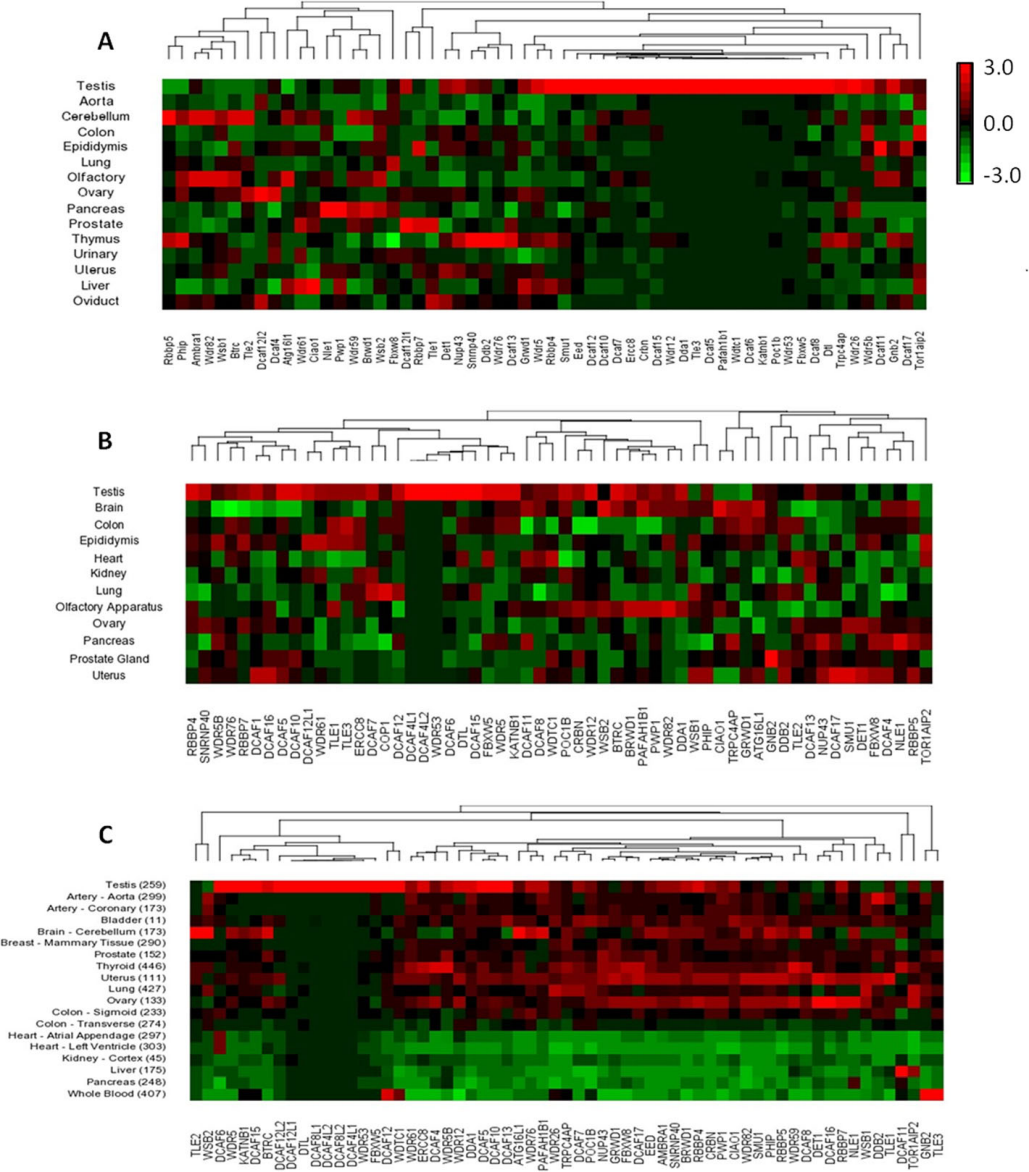
Unsupervised hierarchical clustering of mRNA expression profile of the DCAF genes subfamily across different tissues of mouse and human. Unsupervised hierarchical clustering analyses of mouse **a** and human **b**, **c** DCAF genes using FANTOM5 RNA seq dataset **a**, **b** and GTEx RNA seq dataset **c** showed differential mRNA expression profiles in different tissues of adult mouse and human. Samples are denoted in rows and genes are denoted in columns. Red and green denote highly and weakly expressed genes, respectively

Gene expression profile can provide important clues about the gene function. To investigate the mRNA abundance profiles of WD40 family genes, raw RNA seq data of mouse and human were collected from the FANTOM5 and GTEx data portals as described in materials and methods. The RNA seq data analyses of FANTOM5 datasets identified 292 transcripts of WD40 genes out of 347 WD40 genes in mouse and 289 transcripts of WD40 genes out of 349 WD40 genes in human. Whereas, analysis of the GTEx RNA seq dataset from human detected 313 WD40 gene transcripts out of total 349 WD40 genes. Hierarchical clustering and heat map analyses of mouse and human RNA seq datasets showed differential transcript abundance of WD40 genes in various tissues of mouse and human (Fig. 2a, b, c) with significantly large number of WD40 genes were highly or specifically expressed in the testis as compared to other tissues such as lung, heart, liver, brain, kidney, colon, pancreas, prostate, epididymis, ovary, oviduct and uterus (Fig. 2a, b, c). According to the FANTOM5 datasets, the percentages of highly expressed WD40 genes in the mouse and human testes were 59.6 and 64.4%, respectively (Fig. 2a, b). Similarly, the human GTEx dataset showed differential expression patterns of WD40 genes across different tissues with significantly large number of WD40 genes (73.5%) was highly or specifically expressed in the testis (Fig. 2c). Further analyses of mouse and human FANTOM5 RNA seq datasets detected 58 each transcripts of DCAF subfamily genes out of total 60 and 65 DCAFs in mouse and human, respectively. Hierarchical clustering and heat map analyses of the 58 DCAF genes from each of mouse and human RNA seq datasets displayed differential expression patterns across different tissues of both the species with considerably higher proportion of DCAF genes showed high abundance of transcripts in testis as compared to other tissues analysed in this study (Fig. 3a, b). Around 74 and 83% DCAF genes in mouse and human, respectively, showed specific or notably high expression in testis as compared to other tissues examined (Fig. 3a, b). In concordance, analysis of the GTEx dataset of human DCAF genes also displayed differential expression patterns with large fraction (~ 83%) of the genes were highly or specifically expressed in the testis (Fig. 3c). Based on DCAF gene transcripts abundance in testis, a clear grouping of highly or specifically expressed DCAFs and weakly expressed DCAFs was observed (Fig. 3a, b, c). Among the highly or specifically expressed DCAF genes in the testes of mouse and human, 41 genes were common, suggesting conserved roles in both species.

### DCAF subfamily genes are differentially expressed across the major tissues of mouse and human

To validate the RNA seq data, semi-quantitative RT-PCR was performed using gene specific primers for 56 and 57 DCAF subfamily genes of mouse and human, respectively, as described in supplementary methods. We used 10 and 17 different major tissues from adult mouse and human, respectively, as described in materials and methods. As shown in the Fig. S1A & S1B, all the DCAF genes are differentially expressed across different tissues of mouse and human, respectively. In the mouse testis, the mRNA transcripts for majority the DCAF genes were detected from very low to very high levels, with very few genes showed undetectable levels (Fig. S1A). Some of the DCAF genes like *Gnb2*, *Rbbp7*, *Wdr26*, *Sum1, Wsb1, Nil1* and *Eed* showed almost equal level of transcripts among all the tissues analysed, suggesting that they may have common functions that are required for all the tissues (Fig. S1A). The mRNA transcript levels of the genes *Ddb2, Ercc8, Dcaf5, Dcaf10, Det1, Katnb1, Wdr51, Wdr51b, Wdr12, Tle3* and *Nup43* were highest in testis with moderate to very low levels in other tissues, implying specific or major roles of these genes during spermatogenesis (Fig. S1A). Interestingly, the *Cdt2* gene transcripts were only detected in testis and ovary, suggesting specific and unique role of this gene in gametogenesis (Fig. S1A). Similar expression patterns of human DCAF genes were observed in different human tissues used for this study (Fig. S1B). The *β-actin* gene and 18S rRNA were used as a house keeping genes as well as loading controls showing equal levels of transcripts among all the tissues analysed (Fig. S1A, B).

**Fig. 4:**
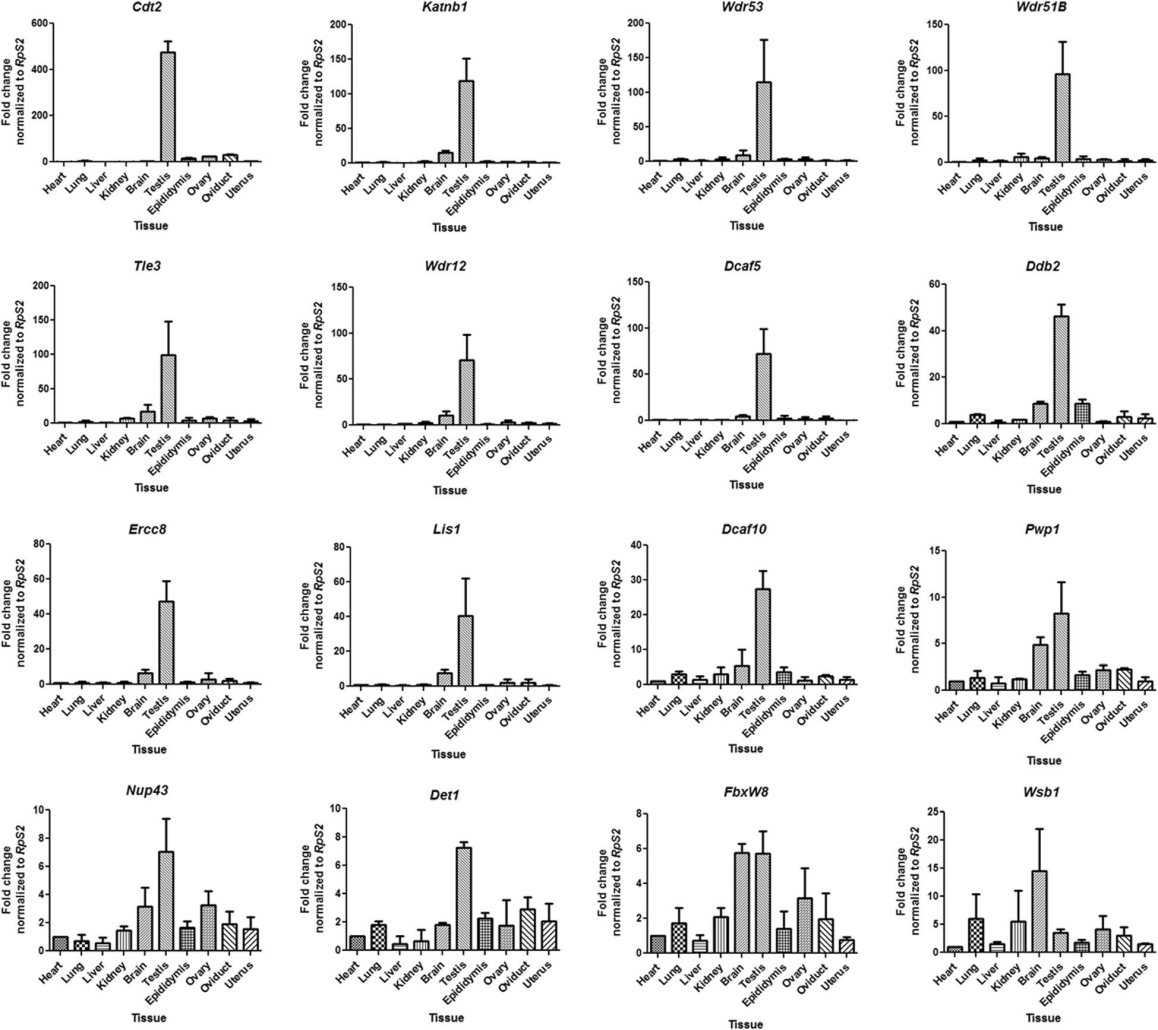
Relative expression profiles of different DCAF subfamily genes across different tissues of adult mouse as revealed by qRT-PCR. Relative expression of 16 different DCAF genes mRNAs across 10 different tissues of adult mouse was calculated relative to heart. *RpS2* was used as an internal control to normalize the data. Y-axis shows relative mRNA expression levels and X-axis shows different tissues. The error bars represent the standard deviation of three technical replicates for each biological triplicate

We further selected 16 of the highly expressed mouse DCAF genes to confirm their expression by qRT-PCR analysis in 10 different tissues from adult mice. The qRT-PCR results confirmed high mRNA levels of the selected DCAF genes in the mouse testis compare to other tissues analysed (Fig. 4). Genes *Wdr12, Wdr51b, Wdr53, Katnb1, Lis1, Tle3, Cdt2, Ddb2, Ercc8, Dcaf5* and *Dcaf10* showed very high levels of transcripts ranging from 28 to 450 folds high in the testis in comparison to other tissues (Fig. 4). Very high levels of transcript in testis suggest that those genes may have crucial and specific roles in spermatogenesis. Other genes *Pwp1, FbxW8, Det1* and *Nup43* showed transcripts abundance ranging from 6 to 14 folds high in the testis while other tissues like brain, ovary and oviduct showed moderate to high transcript levels suggesting that these genes may have shared functions in these tissues (Fig. 4). Expression profiling of the *Wsb1* gene across different tissues showed higher expression in brain followed by lung and kidney than testes (Fig. 4).

### Expression of DCAF subfamily genes during development of the mouse testis

**Fig. 5:**
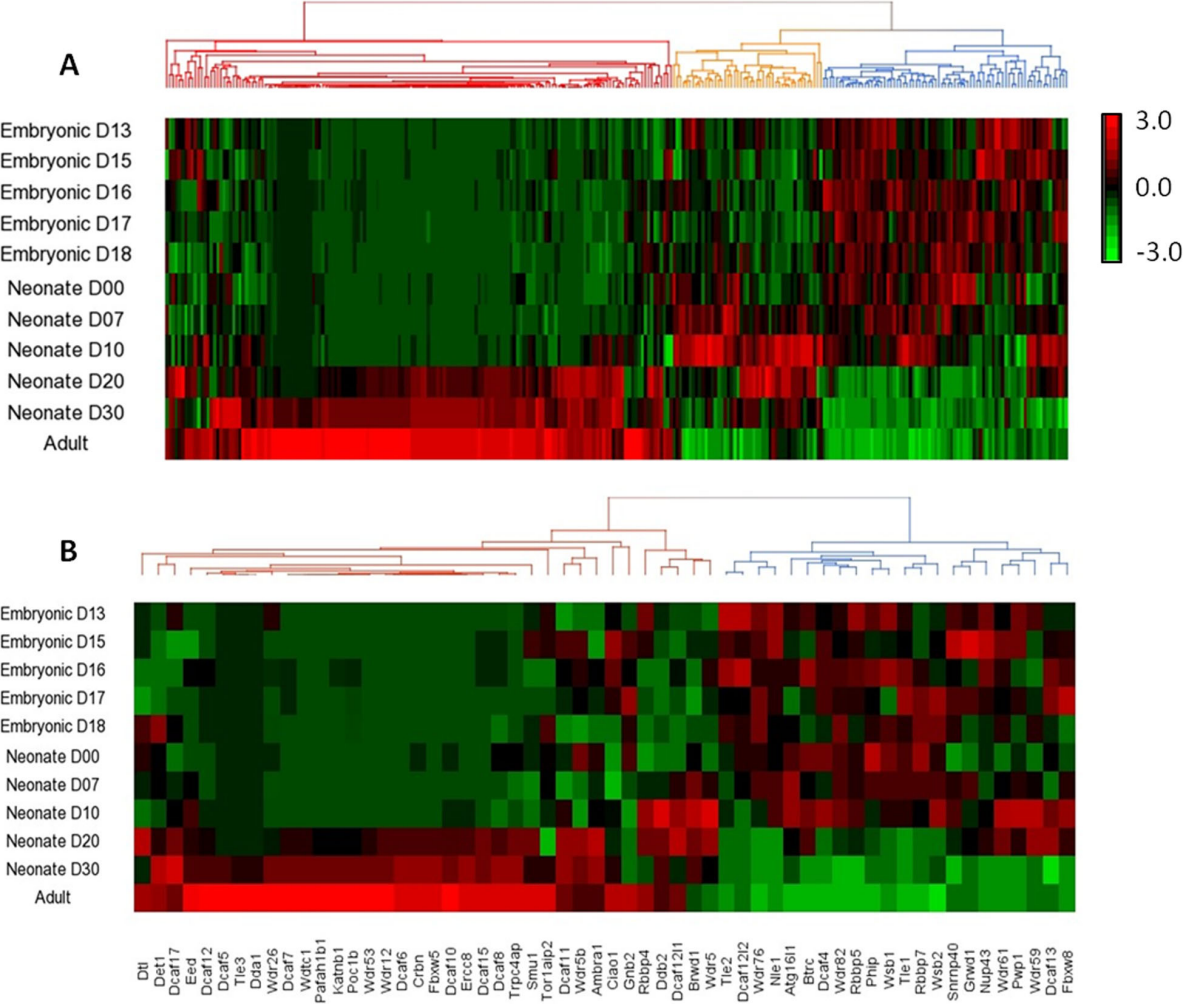
Unsupervised hierarchical clustering of mRNA expression profiles of mouse WD40 encoding genes family **a** and DCAF genes subfamily **b** during different stages of testis development. Unsupervised hierarchical clustering analyses of mouse WD40 encoding genes **a** and DCAF genes **b** using FANTOM5 RNA seq dataset showed differential mRNA expression patterns of the genes during different stages of testis development from embryonic stage to adult mouse. Samples are denoted in rows and genes are denoted in columns. Red and green denote highly and weakly expressed genes, respectively

To explore the mRNA levels of mouse WD40 family and DCAF subfamily genes during normal testicular development, RNA seq data for mouse testes at different developmental stages including embryonic (day 13, 15, 16, 17, and 18), neonate (day 0, 07, 10, 20, and 30), and adult were collected from FANTOM5 repository and analysed. Mining of FANTOM5 dataset from different developmental stages of the mouse testis detected 283 transcripts of WD40 genes. Hierarchical clustering and heat map analyses of the WD40 family and DCAF subfamily genes during different stages of testis development showed diverse expression patterns (Fig. 5a, b). As shown in Fig. 5, varying mRNA levels of WD40 family and DCAF subfamily genes are not only associated with different tissues, but also with the different developmental stages of the testis. According to the expression profiles during different stages of testicular development, the WD40 genes can be classified into three distinct groups. The largest group (159 genes) was highly expressed during post-natal testicular development from day 20 to adult (Fig. 5a). The second group containing 77 genes showed higher expression during embryonic day 13 through neonatal day 10 (Fig. 5a). The third group of 47 WD40 genes is highly enriched during neonatal day 0 through neonatal day 20 after birth (Fig. 5a). Similarly, the DCAF subfamily genes can be classified into two major groups depending upon their expression patterns as observed in the Fig. 5b. The first group of 36 genes showed high levels of transcripts during post-natal development of testis from neonatal day 20 through adult (Fig. 5b). Whereas, the second group of 22 genes showed high levels of transcripts from embryonic day 13 to post-natal day 20 of the testis development (Fig. 5b).

### Stage-specific expression of WD40 family and DCAF subfamily genes during mouse spermatogenesis

**Fig. 6:**
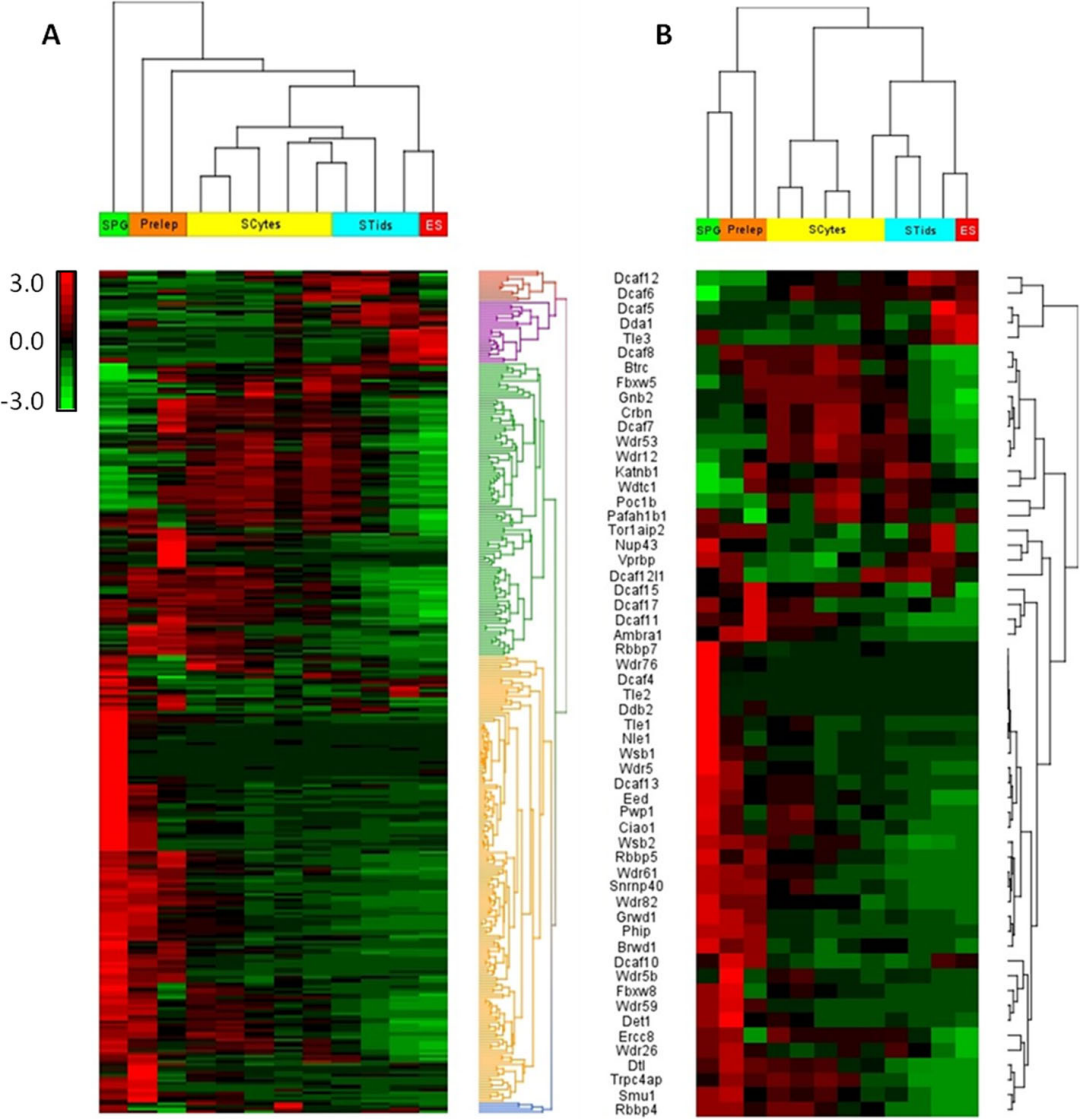
Unsupervised hierarchical clustering of mRNA expression profiles of mouse WD40 encoding genes family **a** and DCAF genes subfamily **b** in different spermatogenic cell populations. Unsupervised hierarchical clustering analyses of mouse WD40 encoding genes **a** and DCAF genes **b** using RNA seq dataset from Green et al.*,* 2018 (Accession: GSE112393) showed differential mRNA expression patterns of the genes in different populations of spermatogenic cells from the mouse testis. Samples are denoted in columns and genes are denoted in rows. Red and green denote highly and weakly expressed genes, respectively. SPG - spermatogonia; Prelep - preleptotene; SCytes - meiotic spermatocytes; STids - post-meiotic haploid round spermatids; ES - elongating spermatids

To further investigate the expression profile of WD40 family and DCAF subfamily genes in different types of germ cell populations in the mouse testis, we used RNA seq datasets derived from Green et al. (Accession: GSE112393) and da Cruz et al. (Accession: PRJNA317251) [50, 51]. Independent mining of RNA seq datasets from Green et al. and da Cruz et al. identified 302 and 255 transcripts of total 347 WD40 genes, respectively. Hierarchical clustering and heat map analyses of the WD40 family and DCAF subfamily genes expressed in different germ cell populations of the mouse testis revealed diverse expression patterns among different spermatogenic cell populations (Fig. 6a, b & S2). Based on the heat map clustering analysis derived from Green et al. data, the WD40 family genes can be classified into three major groups according to their expression patterns in various germ cell populations (Fig. 6a). The first group of the WD40 genes that contained 164 genes were highly expressed in spermatogonia and preleptotene spermatocytes during mitotic stage (Fig. 6a). Second group included 105 genes that showed elevated expression during meiotic stage (Fig. 6a). Finally, the third group of 33 genes showed increased expression in post-meiotic round and elongated spermatids (Fig. 6a). Further analysis of Green et al. dataset for DCAF subfamily genes detected 57 DCAF transcripts with differential expression patterns across different spermatogenic cell populations (Fig. 6b). Depending upon the abundance of DCAF genes mRNA levels and spermatogenic cell populations, hierarchical clustering and heat map analyses of the 57 DCAF genes showed distinct clustering into three major groups that included 40 pre-meiotic, 12 meiotic and 5 post-meiotic genes (Fig. 6b). Similarly, hierarchical clustering and heat map profiling of WD40 gene transcripts from da Cruz et al. dataset displayed distinct expression patterns with four clear clusters based on mRNA abundance in different testicular cell populations (Fig. S2A). The WD40 genes with high abundance of mRNA in spermatogonia and somatic cells (2C) included 50 genes (Fig. S2A). Around 107 genes showed higher expression in meiotic prophase I early stages (leptotene and zygotene; LZ) cell populations (4C). The third cluster contained 44 genes showing increased levels of mRNA in pachytene spermatocytes (PS) (4C) (Fig. S2A). In the round spermatid (RS) population (C), 54 genes were highly expressed as compared to other spermatogenic cells (Fig. S2A). Hierarchical clustering and heat map analyses of DCAF subfamily genes from da Cruz et al. RNA seq dataset also exhibited differential expression patterns of various DCAF gene transcripts across different testicular cell populations (Fig. S2B). Around 18 (~ 30%) of DCAF subfamily genes were predominantly or specifically expressed in spermatogonia and somatic cell populations (2C) (Fig. S2B). More than a half number of DCAF genes (~ 53%) were highly or specifically expressed in leptotene and zygotene (LZ) spermatocyte cell populations (4C) (Fig. S2B). Pachytene spermatocyte (PS) population (4C) showed high or specific expression of around 11 DCAF genes (~ 18%) (Fig. S2B). The number of DCAF genes with high mRNA abundance in round spermatids (RS) population (C) included twelve (~ 20%) genes (Fig. S2B).

Further, hierarchical clustering and heat map analysis of human WD40 family genes and DCAF subfamily genes using Guo et al. RNA seq dataset also showed differential expression patterns in different spermatogenic cell populations (Fig. S3A, B) [52]. According to the levels of WD40 and DCAF genes transcripts in different spermatogenic cells, the target genes can be grouped into three major clusters (Fig. S3A, B). First group of the genes was predominantly expressed in spermatogonia. Second group of the genes showed high transcript levels in spermatocytes and third group of the genes showed high transcript levels in spermatids (Fig. S3A, B).

### qRT-PCR confirmed differential expression patterns of DCAF subfamily genes during different stages of post-natal testis development in mouse

**Fig. 7:**
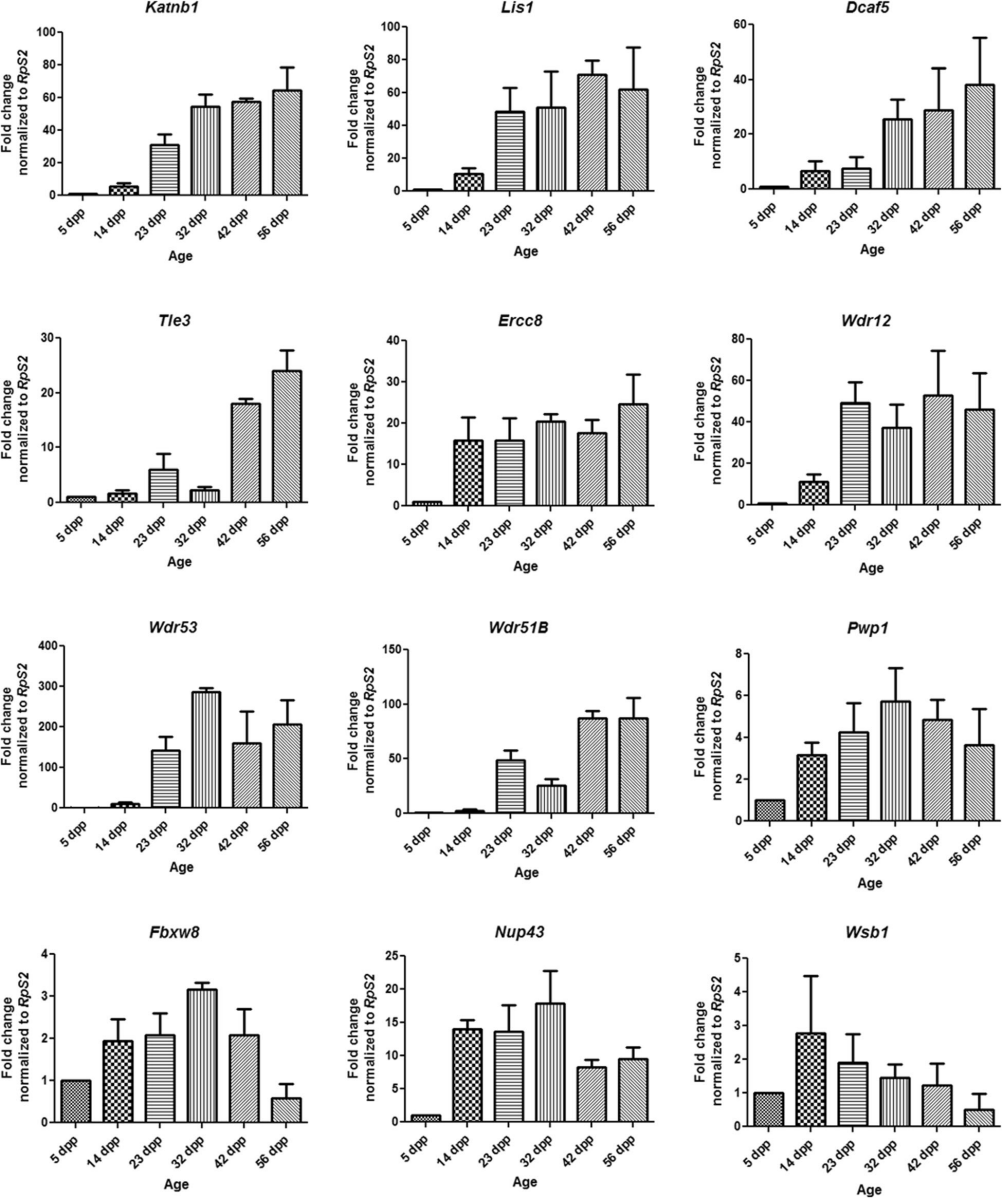
Relative expression profiles of transcript levels of different DCAF subfamily genes during different postnatal developmental stages of the mouse testis as revealed by qRT-PCR. Relative levels of the transcripts of 12 different DCAF genes in the mice testes during different post-natal development stages from 5 to 56 days postpartum (dpp) was calculated relative to 5 dpp. *RpS2* was used as an internal control to normalize the data. Y-axis shows relative mRNA expression levels and X-axis shows different age of the mice from 5 to 56 dpp. The error bars represent the standard deviation of three technical replicates for each biological triplicate

**Fig. 8:**
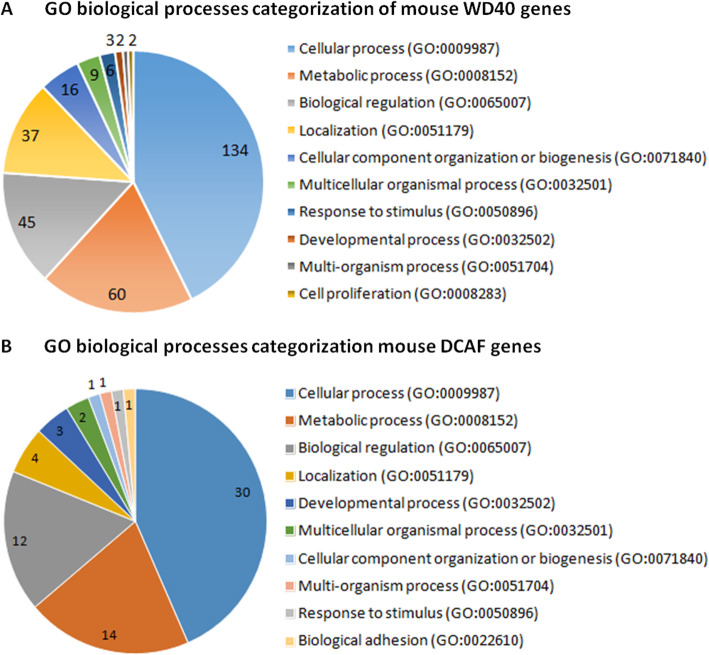
Pie charts showing the gene ontology (GO) categorization of the mouse WD40 family genes **a** and DCAF subfamily genes **b** according to biological processes. WD40 and DCAF genes of mouse were classified into 10 GO categories for biological processes using PANTHER. Size of the pie slice corresponds to the number of genes in a given GO category

To validate the observed differential expression patterns of DCAF subfamily genes in the RNA seq datasets, we randomly selected 12 of the highly expressed DCAF genes in the testis and checked their mRNA levels in mouse testes collected at different ages of 5, 14, 23, 32, 42 and 56 days postpartum (dpp) using qRT-PCR technique. As shown in the Fig. 8, all the selected DCAF genes showed diverse expression patterns during post-natal testis development. Depending upon the expression patterns, DCAF genes can be divided into three broad categories. In first category, low gene expression during early stage of testis development followed by gradual increase in the expression with the age till maturation and then the expression remains constant. The genes in this category include *Dcaf5, Ercc8, Katnb1, Lis1, Tle3, Wdr12, Wdr51B* and *Wdr53* (Fig. 7). Second category of the genes show gradual increase in the transcript levels with the age till certain age, then it decreases until it reaches minimum level at the adult age. Examples of the genes in second category are *Pwp1*, *FbxW8* and *Nup43* (Fig. 7). The gene expression pattern in the third category, which includes *Wsb1*, shows higher expression during early developmental stages of testis and then the gene expression decreases gradually with the advancing age (Fig. 7).

### Gene ontology (GO) annotation and network analysis of WD40 proteins

Gene ontology (GO) classification and enrichment analyses of human and mouse WD40 family and DCAF subfamily proteins were performed using PANTHER [53, 54], DAVID Bioinformatics Resources [55] and Ingenuity Pathways Analysis (IPA). According to PANTHER classification, broad molecular functions were assigned for WD40 and DCAF proteins. Based on the assigned GO terms for biological processes, the results showed putative participation of WD40 proteins in diverse biological processes that were classified into ten major categories (Fig. 8a & S4A, Table S3). Among different categories of biological processes, the WD40 proteins of mouse and human were predominantly participated in cellular processes (~ 130), followed by metabolic processes (~ 60), biological regulation (~ 45) and localization (~ 36) (Fig. 8a & S4A). Similarly, biological function analysis of DCAF proteins from mouse and human using PANTHER classification system classified the DCAF proteins into 10 and 9 biological processes, respectively (Fig. 8b & S4B). Majority of the proteins were involved in cellular process (~ 44%), metabolic process (~ 20%), biological regulation (~ 20%), localization (~ 5%) and developmental process (4%) (Fig. 8b & S4B).

GO enrichment analyses of WD40 family proteins using DAVID analysis revealed more than 290 significant biological process categories, around 100 cellular component categories and around 70 molecular function categories for mouse and human (Fig. S5, Table S4). Genes related to protein ubiquitination (*p*-value = 4.07 × 10^− 15^), cell cycle (*p*-value = 3 × 10^− 8^), protein modification process (*p*-value = 2 × 10^− 7^) and metabolic process (*p*-value = 1.4 × 10^− 3^), were among the most significantly enriched terms (Table S4). Likewise, the GO enrichment analysis of DCAF subfamily proteins recognized around 70 biological process categories, 25 cellular component categories and around 25 molecular function categories for both species (Fig. S6, Table S5). Genes related to protein ubiquitination (p-value = 7.83 × 10^− 18^), cellular protein modification process (*p*-value = 3.4 × 10^− 11^), histone modification (p-value = 6.58 × 10^− 6^) and covalent chromatin modification (p-value = 3.87 × 10^− 5^) were among the most significantly enriched terms (Table S5).

**Fig. 9:**
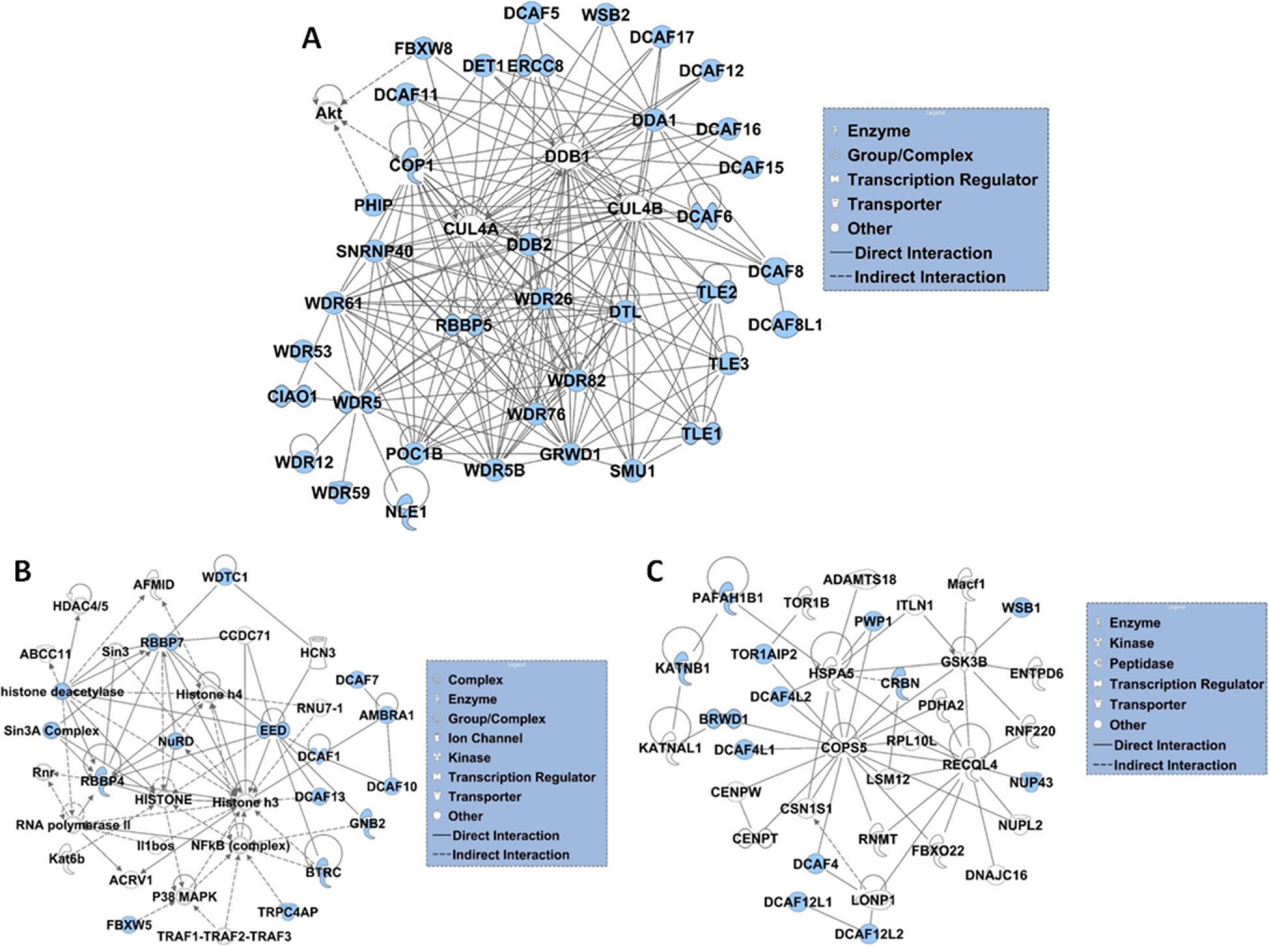
Network analysis of DCAF subfamily genes. Three significant interaction networks predicted by the ingenuity pathway analysis (IPA) software (QIAGEN Inc., https://www.qiagenbioinformatics.com/products/ingenuity-pathway-analysis) are presented. **a** Network of proteins associated with ubiquitination. **b** Network of proteins associated with transcription, chromatin modification, sperm maturation, sperm motility and histone modifications. **c** Network of proteins involved in ubiquitination, microtubule dynamics, transcription regulation, Wnt signalling pathway and spermatogonia differentiation. The shapes represent functional class of the genes product, as indicated in legend. Colored nodes are genes from the input list of DCAF genes and uncolored genes are predicted by ingenuity pathway knowledge data base as the interactors. Straight and dashed lines represent direct or indirect interactions, respectively

The DCAF proteins were further subjected to interaction network analysis by IPA. The top three scoring networks were shown in Fig. 9a-c. The first network included 41 proteins associated with post-translational modification, especially ubiquitination. The second network comprised of 36 proteins that are mainly involved in transcription, chromatin modification, sperm maturation, sperm motility and histone modifications. The third network included 34 proteins that are involved in ubiquitination, microtubule dynamics, transcription regulation, Wnt signalling pathway and spermatogonia differentiation. The first network was characterized by presence of genes involved in protein ubiquitination, DNA damage response and repair and global genomic repair (Fig. 9a, Table S6). The genes in network 1 were mainly linked to different types of cancers and loss of germ cells (Table S6). Network 2 was mainly associated with transcription, chromatin remodelling, apoptosis, cell cycle and protein ubiquitination (Fig. 9b, Table S6). The genes in network 2 were found to be linked with various types of cancers (Table S6). Network 3 was associated with organelle assembly, microtubule morphology, nuclear transport and protein ubiquitination (Fig. 9c, Table S6).

## Discussion

To gain comparative insights into the genome-wide transcriptional signatures of WD40 family genes in multiple tissues of mouse and human, we systematically mined and investigated their relative transcript levels using in silico and qRT-PCR techniques with specific focus on testicular expression profiling of DCAF subfamily genes. We identified more than 345 putative WD40 protein encoding genes including DCAFs from each mouse and human genomes and examined their expressions in different tissues as well as in different developmental stages of testis from several high-throughput gene expression datasets. In earlier studies using SMART database, it has also been predicted to have 349 proteins containing WD40 domain in the human [3, 4, 56]. Comprehensive in silico study of the human WD40 family by Zou et al. identified 262 non-redundant WD40 protein encoding genes [14]. Similarly, sequence search-based domain analysis of protein database identified 265 WD40 protein coding genes in mouse genome [13]. The discrepancy between the number of WD40 encoding genes identified in human genome by Zou et al. and in this study is because the study by Zou et al. have considered only WD40 proteins that contain six or more WD40 repeats [14]. Whereas, in our study we have included all the proteins containing one or more WD40 repeats. Variations observed in the numbers of predicted WD40 genes among previous studies and this study may be due to the low level of sequence similarities among WD40 repeats, the variable number of repeats within a single WD40 domain, and functional diversity of WDR proteins [57].

Analyses of different mouse and human RNA seq datasets revealed substantial variation in the abundance of WD40 gene transcripts among different tissues of both the species, indicating their diverse and tissue specific roles in different cellular and developmental processes. Previous studies have reported many WD40 genes that are not only involved in different cellular and developmental processes but they are also linked to diseases [3–5, 9]. We found that significantly large number of WD40 genes (~ 60–73%) was predominantly or specifically expressed in the testis of mouse as well as human, suggesting that these genes may have important functions in testicular development and/or spermatogenesis. Our findings are supported by earlier study on RNA seq data analysis from the Human Protein Atlas by Zou et al. where it was shown that the testis expressed highest number of tissue-specific WD40 genes [14]. Similarly, microarray data analysis of silkworm WD40 gene family identified a large number of these genes that were highly or specifically expressed in testis [13]. Expression studies on some of the WD40 genes such as *Lis1 (Pafah1B1), wuho, Wdr5 (Big-3), Wsb2, Wdc146, Wdr73, Wdr13, Crbn, Dcaf17* and *Tle3* in different species by different research groups also showed significantly higher expression of those genes in the testis as compared to other tissues [39–41, 46, 58–63]. In addition, functional and genetic studies conducted by various groups on several WD40 family genes (*β-TrCP, Katnb1, Ddb2, Brwd1, Dcaf17* and *Lis1*) also showed that these genes play critical roles in different stages of testis development, spermatogenesis and fertility [42–46, 64–67]. For example, human mutations and knockout mouse models for Cilia and flagella associated protein (CFAP) 43 and 44 (members of WD40 gene family) showed similar defective sperm morphology, sperm motility and male infertility [68]. Moreover, male *Dcaf17* knockout mouse model exhibited lower sperm count and motility, abnormal seminiferous tubules and disorganized sperm head structure [46]. Mutations in *DCAF17* gene caused hypogonadism among other symptoms including diabetes and mental retardation in human [12]. The aforementioned genes are highly expressed in the testis of both species which underline the importance of elucidating contributions of WD40 genes in male fertility.

Similarly, the RNA seq dataset analyses for DCAF subfamily genes, a subgroup of WD40 family genes, of mouse and human also showed significant variation in the amount of each gene transcripts within various tissues with noticeably higher proportion (~ 74–83%) of DCAF genes were highly or specifically expressed in the testis as compared to other tissues in both, mouse and human. These were further confirmed by RT-PCR/qRT-PCR results in this study. Our findings suggest that the DCAF genes may play different and critical roles during the testicular development and spermatogenesis. In addition, analyses of RNA seq datasets of the mouse testes developmental stages revealed that all the WD40 and DCAF genes showed variable and specific expression patterns at different developmental stages. Based on the expression patterns of WD40 genes during testis development, the genes can be clearly divided into three major groups where a set of the genes was highly expressed during embryonic stage, second set of the genes was highly expressed during post-natal testis development stages from day 0 to day 20, and third set of the genes was highly expressed during post-natal testis developmental stages from day 20 to adult. Likewise, expression patterns of DCAF subfamily genes showed two major and distinct groups with one set of the genes was predominantly expressed during testis embryonic development and the second set of the genes was highly expressed during post-natal testicular development from neonatal day 20 to adult. These diverse expression characteristics of WD40 and DCAF genes, during testis development suggest that encoded proteins may regulate a complex network of pathways to perform different cellular and molecular functions during testicular development process. The *Wsb2* gene expression study in mouse embryonic and adult gonads showed expression of the *Wsb2* in the developing male mouse gonads at 11.5 dpc with gradual increase in the expression during gonadal development [41]. Similarly, mouse *Wdr13* gene exhibited expression during early stages of gonadal development through adult testis with a predominant expression in the germ cells of adult testis [63]. Another study on *Wdr73* expression in *Larimichthys crocea* demonstrated that the *Wdr73* expression increased with the advancement of gonad development, starting from proliferative stage to mature stage [62].

Spermatogenesis is a highly regulated, dynamic and intricate biological process in which undifferentiated diploid spermatogonia undergo self-renewal and proliferation by number of mitotic divisions, two meiotic divisions and spermiogenesis to produce highly specialized mature spermatozoa [37, 69]. It is controlled by a synchronized expression of defined sets of genes in a stage-specific manner [50, 51]. Investigation of the expression patterns of all WD40 genes and separately of DCAF subfamily genes only showed differential expression patterns in different spermatogenic cell types, suggesting involvement of these genes in a cell- and stage-specific manner during spermatogenesis. Quantitative RT-PCR results examining testis highly expressed DCAF subfamily genes expression during post-natal testis development confirmed the differential and stage-specific expression patterns of these genes. This varying expression of DCAF genes during testicular development suggests that their expression is developmentally regulated to maintain normal spermatogenesis. In mammals, spermatogenesis begins from the day of birth. However, actively proliferating type A spermatogonia and Sertoli cells could be recognized from postnatal day 5 or 6 in mice [70–72]. The seminiferous epithelium of 5 to 6 days old mice testes contains only primitive type A spermatogonia and Sertoli cells. The type A spermatogonia undergo a series of mitotic division and give rise to type B spermatogonia by day 8. Around day 9 or 10, the type B spermatogonia enter meiotic division by generating primary spermatocytes. As the meiotic cell division progresses, primary spermatocytes produce early and late pachytene stage spermatocytes by days 14 and 18, respectively. Around day 18 of development, pachytene stage spermatocytes complete first meiotic division to form secondary spermatocytes, which subsequently undergo second meiotic division to produce haploid round spermatids at the onset of spermiogenesis on around day 20. By day 35 first cycle of spermatogenesis is completed [70–72]. The biochemical and morphological changes in germ cells during spermatogenesis require highly regulated, balanced, spatiotemporal expression of the genes, which presumably reflect differences in the mRNA populations of different stage-specific DCAF genes during germ cell development. DCAF proteins are proposed to function as substrate receptors for CRL4 complexes [23]. Hou et al. have identified large number of highly or specifically expressed E3 ligases in mouse testis in a cell- and stage-specific manner [49]. Proposed role of DCAF proteins in CRL4 mediated ubiquitination and similarity between the differential expression patterns of DCAF genes and E3 ligase genes suggest that proteins encoded by these genes may play critical roles in the regulation of spermatogenesis through ubiquitination of target proteins [23]. However, future functional studies still need to be conducted to show testicular role for such genes with unknown male reproductive phenotypes. Dependence of spermatogenesis on proteolytic and non-proteolytic actions of protein ubiquitination is well documented and many E3 ligases have be shown to play vital roles in many of the cellular and molecular process required for the production of mature spermatozoa [33, 73, 74].

To know the significance of WD40 family genes, we performed comprehensive bioinformatics analyses using PANTHER, DAVID and IPA software. GO annotation and enrichment analyses of WD40 family and DCAF subfamily genes revealed that the top over-represented functions were related to protein ubiquitination, cellular processes, metabolic processes, biological regulations and localization, suggesting functional diversity of WD40 proteins. Molecular network modelling of DCAF subfamily genes using IPA, identified three top ranking molecular networks related to post-translational modification, chromatin modification, sperm maturation and motility, microtubule dynamics, transcription and spermatogonia differentiation, suggesting importance of the DCAF subfamily genes in the regulation of testicular development and spermatogenesis.

## Conclusions

We performed comprehensive and systematic mining, and expression profiling of the putative WD40 protein encoding genes in different tissues of mouse and human. Our investigation identified 347 and 349 WDR protein encoding genes in the genomes of mouse and human, respectively. High throughput RNA seq datasets analyses of WD40 genes in mouse and human revealed differential expression patterns among different tissues with a significantly large number of these genes were highly or specifically expressed in testis, suggesting their distinct and tissue specific functions in testicular development and spermatogenesis. A subset of WD40 genes called DCAF subfamily genes that encode substrate receptor proteins for CRL4 complexes. We identified 60 and 65 DCAF genes in mouse and human genomes, respectively. RNA seq and RT-PCR/qRT-PCR analyses of DCAF genes revealed diverse expression patterns of these genes across different tissues with considerably large proportion of the genes were predominantly or specifically expressed in the testis of both the species. Further analyses of WD40 and DCAF genes expression during different stages of embryonic and post-natal testis development in mouse and also in different spermatogenic cell populations revealed developmental and spermatogenic stage-specific regulation of these genes. Together, our data implicated intricate expression patterns for WD40 genes, particularly DCAF genes, reflecting complexity of spermatogenesis and also provide a valuable source to further elucidate their functions during spermatogenesis. Information gained from this study will help to develop infertility interventions and/or male-specific contraception tools.

## Methods

### Data collection and mining

A comprehensive search for WD40 domain protein encoding genes including DCAF subfamily genes of mouse and human was conducted using the Mouse Genome Informatics (MGI) [http://www.informatics.jax.org/], Human Genome Organization (HUGO), HUGO Gene Nomenclature Committee (HGNC) [https://www.genenames.org/data/genegroup/#!/], Ensembl [https://www.ensembl.org/index.html] and Ingenuity® Pathway Analysis (IPA) (QIAGEN, www.qiagen.com/ingenuity) databases and literature [10, 13, 14, 23–25, 75–79]. The *WD40, DCAF, DWD, CDW, DDB1/2* and *CUL4* terms were used in our search to retrieve the list of WD40 family genes. The RNA sequencing (RNA seq) based high-throughput gene expression data of the genes encoding WD40 family proteins in different tissues of mouse and human were obtained from the publically available RNA seq datasets from data resources and projects: The Functional ANnoTation Of Mammalian genomes (FANTOM5) [http://fantom.gsc.riken.jp/5/] (with Accession #s E-MTAB-3579 and E-MTAB-3358 for mice and human tissues, respectively), and Genotype-Tissue Expression (GTEx) [https://gtexportal.org/home/] [80–85]. Normalized tags (counts) per million (TPM) were computed using the relative log expression (RLE) algorithm in the FANTOM5 dataset. In the GTEx dataset, the expression values were normalized between samples using the trimmed mean of M-values (TMM) normalization method. Gene expression data for mouse and human in FANTOM5 datasets were used to investigate the expression patterns of the genes encoding WD40 family proteins in 15 tissues of adult mice (testis, aorta, cerebellum, colon, epididymis, lung, olfactory apparatus, ovary, pancreas, prostate, thymus, urinary bladder, uterus, liver and oviduct) and 10 human tissues (testis, brain, colon, epididymis, lung, olfactory apparatus, ovary, pancreas, prostate gland and uterus). For mouse testicular tissue, gene expression data for several testicular developmental stages that cover embryonic (day 13, 15, 16, 17, and 18), neonate (day 0, 07, 10, 20, and 30), and adult were also investigated. The RNA seq data for different types of spermatogenic cells from mouse testis were obtained from the public functional genomics data repository “Gene Expression Omnibus (GEO)” [Accession: GSE112393] [50] and “NCBI Sequence Read Archive (SRA)” [Accession: PRJNA317251] [51].

### Animal use and tissue collection

C57BL/6 J mice at different ages [5, 14, 23, 32, 42 and 56 dpp] were used for this study. All the mice were obtained from the King Faisal Specialist Hospital and Research Centre laboratory animal services, Riyadh, Saudi Arabia. The mice were housed under controlled conditions of temperature (21 ± 1 °C), humidity and a 12 h light/12 h dark cycles. Animals were provided free access to standard rodent chow and tap water. All the procedures for animal care and use were approved by the Institutional Animal Care and Use Committee (IACUC) at King Faisal Specialist Hospital and Research Centre, Riyadh (Project RAC# 2170 024). Procedures for animal euthanasia and tissue collection were carried out according to the IACUC and National Research Council’s Guide for the Care and Use of Laboratory Animals. All the animals above ten days old were sacrificed by cervical dislocation, while 10 days old and younger mice were euthanized by decapitation before tissue collection. Seven tissues (brain, heart, lung, liver, kidney, testis and epididymis) were dissected from 56 dpp old adult male C57BL/6 J mice and three tissues (ovary, oviduct and uterus) were dissected from 56 dpp old adult female C57BL/6 J mice. Testes were separately collected from the 5, 14, 23, 32 and 42 dpp old mice. Testes of 5 dpp and 14 dpp old mice were pooled from five animals each to yield sufficient total RNA samples. Triplicate tissue samples were obtained in a similar fashion from the same breeding pairs. Tissues were snap frozen in liquid nitrogen and placed at − 80 °C until the time of total RNA isolation.

### Total RNA extraction and reverse transcription

Total RNAs from mouse tissues (brain, heart, lung, liver, kidney, testis, epididymis, ovary, oviduct, and uterus) were purified using TRIzol® reagent (Invitrogen, Life Technologies, Waltham, MA, USA), as per company’s provided protocol. Quality and quantity of the purified RNA samples were measured by absorbance at 260 and 280 nm using NanoDrop 2000c spectrophotometer (ThermoFisher Scientific, Waltham, MA, USA). Integrity of purified RNA samples was checked by electrophoresing the RNA samples on 2% denaturing Tris-acetate-EDTA (TAE) ethidium bromide stained agarose gel. Only those RNA samples showing an OD_260_/OD_280_ ratio between 1.8 to 2.1 and intact 28S and 18S RNA bands were used for cDNA synthesis. In case of human samples, the Total Human RNA Master Panel II and Human Ovary Total RNA (Clontech Laboratories, Incorporation, Mountain View, CA) of total RNA samples from selected tissues (Brain, heart, lung, liver, stomach, kidney, thymus, small intestine, skeletal muscles, bone marrow, spinal cord, colon, prostate, testis, ovary, uterus and placenta) were used for cDNA synthesis. Approximately 1 μg (for mouse) and 0.5 μg (for human) of purified total RNA samples were reverse transcribed into cDNA in a total of 20 μl cDNA synthesis reaction mixture using Superscript III First Strand Synthesis system (Invitrogen, Life Technologies, Waltham, MA, USA) and BIO-RAD C1000 Touch™ thermal cycler (BIO-RAD, CA, USA) as per manufacturer’s protocol. Oligo (dT) primer was used for cDNA synthesis with the following thermal cycle conditions: 65 °C for 5 min, 50 °C for 50 min, and 85 °C for 5 min, followed by incubation at 37 °C for 20 min after addition of RNaseH enzyme. The total RNA samples from different tissues were processed for cDNA synthesis simultaneously to avoid experimental variation.

### Semi quantitative reverse transcription-polymerase chain reaction (RT-PCR)

The PCR amplifications were performed in 25 μl reaction volumes containing 2.5 μl of 10Χ Qiagen PCR buffer (Tris·Cl, KCl, (NH4)_2_SO_4_, 15 mM MgCl_2_; pH 8.7), 0.5 μl dNTP mix (10 mM each), 2 μl gene specific forward and reverse primers (Table S7) mixture (5 μM each), 0.5 μl MgCl_2_ (25 mM), 3 μl cDNA (1:10 diluted) as a template, 16.3 μl nuclease-free water and 0.2 μl Taq DNA polymerase (25 Units). The PCR reaction mixtures were subjected to amplification cycles on BIO-RAD C1000 Touch™ thermal cycler (BIO-RAD, CA, USA) using the thermal cycler conditions of initial denaturation at 94 °C for 15 min, 35 cycles of denaturation at 94 °C for 30 s, annealing at 59 °C for 30 s and extension at 72 °C for 45 s followed by final extension at 72 °C for 10 min. The PCR amplicons were loaded on 1% denaturing TAE agarose gel pre-stained with ethidium bromide using 5 μl of PCR product mixed with 1 μl 6X loading dye (Promega). To estimate the size of DNA samples 100 bp DNA ladder (Promega) was run on the agarose gel alongside the samples. To visualize PCR amplification product, the gel was exposed to UV light using an ImageQuant LAS 4000 (GE Life Sciences) imaging system and the gel images were captured using ImageQuant LAS 4000 software (GE Life Sciences).

### Quantitative real-time polymerase chain reaction (qRT-PCR)

The qRT-PCR was performed on a 7500 Fast real-time PCR system (Applied Biosystems, CA, USA) using the PowerUp™ SYBR® Green Master Mix (Applied Biosystems, CA, USA). The qRT-PCR reaction mixture for each target gene contained 12.5 μl of 2Χ PowerUp™ SYBR® Green Master Mix, 1 μl of gene specific primer pairs (forward and reverse primers mixture) at final concentrations of 5 μM for each primer, 3 μl of diluted cDNA (dilution 1:10) as template and 3.5 μl of nuclease free distilled water in a total reaction volume of 20 μl. For negative control, cDNA template was omitted and equal volume of nuclease free dH_2_O was added to the reaction. Exon-exon junction spanning RT-PCR primers for target genes were designed using Primer-BLAST tool from NCBI [https://www.ncbi.nlm.nih.gov/tools/primer-blast/] [86]. The sequences of the gene specific primers are shown in Table S7. Primers were designed to obtain an amplified product in a range of 100–300 bps, and their specificity was tested using BLASTn searches (http://www.ncbi.nlm.nih.gov/BLAST/). The ribosomal S2 protein (*RpS2*) or 18S rRNA gene was used as a housekeeping gene. The 7500 Software v2.3 (Applied Biosystems, CA, USA) was used to set up the qRT-PCR experiments. The qPCR cycle conditions were as follows: 1 cycle of activation at 95 °C for 15 min, and 40 cycles of denaturing at 95 °C for 15 s, annealing at 60 °C for 30 s and extension at 72 °C for 45 s, followed by default slow temperature-ramping dissociation steps (95 °C for 15 s, 60 °C for 15 s, and 95 °C for 15 s) for melting curve. The specificity of the PCR reactions was assessed by a melting curve analysis. All the RT-PCR reactions were performed on 3 technical replicates with three biological samples. Relative expression levels of target genes were analysed by the 2^-∆∆CT^ method as previously described [87]. Briefly, cycle threshold values (Ct) for the genes of interest and the *RpS2* or *18S rRNA* gene (housekeeping gene) were determined and collected using the 7500 Software v2.3 (Applied Biosystems, CA, USA). Ct values for the gene of interest were normalized to those of housekeeping gene values in each sample, and then the fold change for the gene of interest was calculated relative to the level in the reference sample. One-way ANOVA was performed, followed by pair wise comparisons of the means at a *p* value of ≤0.01, using Graphpad PRISM5 software (GraphPad Software, San Diego, CA, USA; https://www.graphpad.com/scientific-software/prism/).

### Bioinformatics and data analysis

We performed unsupervised two-dimensional hierarchical clustering using Pearson’s correlation with average linkage clustering for mouse and human samples, separately based on their gene expression similarity using WD40 repeat encoding genes including DCAF family genes. In addition, functional and gene ontology analysis of genes were performed using QIAGEN’s Ingenuity Pathway Analysis (IPA®, QIAGEN Inc., Redwood City, CA, USA, https://www.qiagenbioinformatics.com/products/ingenuity-pathway-analysis), DAVID [55, 88] and PANTHER™ classification systems [53, 54]. *P*-values were determined by applying right-tailed Fisher’s exact test to determine the probability that the biological function assigned to that data set is explained by chance alone.

### Statistical analysis

For comparison of gene expression in testis versus other tissues and gene expression in testis at different ages, one-way ANOVA followed by a Fisher’s protected least significant difference test was performed using GraphPad PRISM version 5 (GraphPad Software, San Diego, CA, USA). A Student’s t test was conducted to compare the difference between two means. Comparison of multiple means was conducted by one-way ANOVA followed by a Bonferroni’s Multiple Comparison Test. The significance level was set at *p* < 0.05.

## Supplementary information

**Additional file 1: Figure S1.** A: Agarose gel images of RT-PCR products of different DCAF genes from mouse (A). RT-PCR was performed on total RNA from different tissues of adult C57BL/6 mouse using gene specific primers for 56 different DCAF subfamily genes as well as housekeeping genes (β-actin and 18S rRNA). The PCR products were separated on 1.5% agarose gel and images were captured using gel imager. gDNA: Genomic DNA; -ve: No template control. **Figure S1.** B: Agarose gel images of RT-PCR products of different Dcaf genes from human (B). RT-PCR was performed on total RNA from different tissues of adult human using gene specific primers for 52 different DCAF subfamily genes as well as housekeeping genes (18S rRNA). The PCR products were separated on 1.5% agarose gel and images were captured using gel imager. gDNA: Genomic DNA; -ve control: No template control. **Figure S2.** Heat map of WD40 family genes (A) and DCAF subfamily genes (B) showing differential expression in different populations of purified spermatogenic cells from mouse testes. Heat map of WD40 and DCAF genes generated from Cruz et al’s RNA sequencing dataset (da Cruz et al., 2016). Samples are denoted in rows and genes are denoted in columns. Red and green denote highly and weakly expressed genes, respectively. LZ - leptotene and zygotene; 2C - spermatogonia, secondary spermatocytes and Sertoli cells; PS - pachytene spermatocytes; RS - round spermatids. **Figure S3.** Heat map of WD40 family genes (A) and DCAF subfamily genes (B) showing differential expression in different populations of purified spermatogenic cells from human testes. Heat map of WD40 and DCAF genes generated from Guo et al’s RNA sequencing dataset (Guo et al., 2018). Samples are denoted in columns and genes are denoted in rows. Red and green denote highly and weakly expressed genes, respectively. 1 – Spermatogonial stem cells (SSCs); 2 – Differentiating spermatogonia; 3 – Early primary spermatocytes; 4 – Late primary spermatocytes; 5 – Round spermatids; 6 – Elongated spermatids; 7 & 8 – Sperm. **Figure S4.** Pie charts showing the gene ontology (GO) categorization of the human WD40 family genes (A) and DCAF subfamily genes (B) according to biological processes. WD40 and DCAF genes of human were classified into 10 GO categories for biological processes using Panther. Size of the pie slice corresponds to the number of genes in a given GO category. **Figure S5.** Pie and bar charts representing the gene ontology (GO) categorization of the mouse(A, C, E) and human (B, D, F) WD40 family genes according to biological processes (A, B), cellular components (C, D) and molecular functions (E, F) using DAVID. **Figure S6.** Pie and bar charts representing the gene ontology (GO) categorization of the mouse (A, C, E) and human (B, D, F) DCAF family genes according to biological processes (A, B), cellular components (C, D) and molecular functions (E, F) using DAVID.

**Additional file 2: Table S1.** List of WD40-repeat encoding genes from mouse genome.

**Additional file 3: Table S2.** List of DCAF subfamily genes in different species.

**Additional file 4: Table S3.** GO categorization for biological processes of mouse WD40 genes using Panther.

**Additional file 5: Table S4.** GO categorization for cellular component of mouse WD40 genes using DAVID.

**Additional file 6: Table S5.** GO categorization for biological processes of human DCAF genes using DAVID.

**Additional file 7: Table S6.** IPA network analysis of DCAF subfamily genes.

**Additional file 8: Table S7.** DCAF family genes specific primer sequences and PCR product size for mouse and human.

## Data Availability

All data generated or analysed during this study are included in this published article and its supplementary information files. The RNA sequencing datasets obtained from publicly available web-based resources and analysed during the current study are available in the Functional ANnoTation Of Mammalian genomes (FANTOM5) [http://fantom.gsc.riken.jp/5/] (Accession # E-MTAB-3579 and E-MTAB-3358), Genotype-Tissue Expression (GTEx) [https://gtexportal.org/home/], Gene Expression Omnibus (GEO) (Accession # GSE112393) [https://www.ncbi.nlm.nih.gov/geo/query/acc.cgi?acc=GSE112393] and NCBI Sequence Read Archive (SRA) (Accession # PRJNA317251) [https://www.ncbi.nlm.nih.gov/bioproject/PRJNA317251/] repositories.

## Abbreviations

ANOVA: Analysis of variance
ATP: Adenosine Triphosphate
BLAST: Basic Local Alignment Search Tool
CDW: CUL4–DDB1-associated WDR
CFAP: Cilia and flagella associated protein
CRL4: Cullin4-RING-based E3 ubiquitin ligase
Ct: Cycle threshold values
CUL4: Cullin 4
DAVID: Database for annotation, visualization and integrated discovery
DCAF: DDB1–CUL4-associated factors
DDA1: DET1 and DDB1 associated 1
DDB1: Damaged DNA Binding 1
DET1: De-etiolated homolog 1
dNTP: Deoxynucleoside triphosphate
dpc: Days postcoitum
dpp: Days postpartum
DWD: DDB1-binding/WD40 domain
E3s: Ubiqitin E3 ligases
EDTA: Ethylenediaminetetraacetic acid
FANTOM5: Functional ANnoTation Of Mammalian genomes 5
GEO: Gene Expression Omnibus
GTEx: Genotype-Tissue Expression
GO: Gene ontology
HGNC: HUGO Gene Nomenclature Committee
HUGO: Human Genome Organization
IACUC: Institutional Animal Care and Use Committee
IPA: Ingenuity® Pathway Analysis
MGI: Mouse Genome Informatics
LZ: Leptotene and zygotene stage of spermatocytes
mRNA: Messenger RNA
NCBI: National Center for Biotechnology Information
PANTHER: Protein ANalysis THrough Evolutionary Relationships
PCR: Polymerase chain reaction
PS: Pachytene spermatocytes
qRT-PCR: Quantitative real-time polymerase chain reaction
RLE: Relative log expression
RNA seq: RNA sequencing
RNA: Ribonucleic acid
rRNA: Ribosomal RNA
RS: Round spermatid
RT-PCR: Reverse transcription-polymerase chain reaction
SMART: Simple Modular Architecture Research Tool
SRA: Sequence Read Archive
TAE: Tris-acetate-EDTA
TMM: Trimmed mean of M-values
TPM: Tags (counts) per million
UV: Ultraviolet light
WDR or WD40: Tryptophan (W)-aspartate (D) 40-repeats
Wnt: Wingless-related integration site
