# Supplementary material for: Expression profiling of WD40 family genes including DDB1- and CUL4- associated factor (DCAF) genes in mice and human suggests important regulatory roles in testicular development and spermatogenesis
Source: BMC Genomics. 2020 Aug 31;21:602. doi: 10.1186/s12864-020-07016-9 (PMC7457511; doi:10.1186/s12864-020-07016-9)
Supplement: Supplementary file 1 — Additional file 1: Figure S1. A: Agarose gel images of RT-PCR products of different DCAF genes from mouse (A). RT-PCR was performed on total RNA from different tissues of adult C57BL/6 mouse using gene specific primers for 56 different DCAF subfamily genes as well as housekeeping genes (β-actin and 18S rRNA). The PCR products were separated on 1.5% agarose gel and images were captured using gel imager. gDNA: Genomic DNA; -ve: No template control. Figure S1. B: Agarose gel images of RT-PCR products of different Dcaf genes from human (B). RT-PCR was performed on total RNA from different tissues of adult human using gene specific primers for 52 different DCAF subfamily genes as well as housekeeping genes (18S rRNA). The PCR products were separated on 1.5% agarose gel and images were captured using gel imager. gDNA: Genomic DNA; -ve control: No template control. Figure S2. Heat map of WD40 family genes (A) and DCAF subfamily genes (B) showing differential expression in different populations of purified spermatogenic cells from mouse testes. Heat map of WD40 and DCAF genes generated from Cruz et al’s RNA sequencing dataset (da Cruz et al., 2016). Samples are denoted in rows and genes are denoted in columns. Red and green denote highly and weakly expressed genes, respectively. LZ - leptotene and zygotene; 2C - spermatogonia, secondary spermatocytes and Sertoli cells; PS - pachytene spermatocytes; RS - round spermatids. Figure S3. Heat map of WD40 family genes (A) and DCAF subfamily genes (B) showing differential expression in different populations of purified spermatogenic cells from human testes. Heat map of WD40 and DCAF genes generated from Guo et al’s RNA sequencing dataset (Guo et al., 2018). Samples are denoted in columns and genes are denoted in rows. Red and green denote highly and weakly expressed genes, respectively. 1 – Spermatogonial stem cells (SSCs); 2 – Differentiating spermatogonia; 3 – Early primary spermatocytes; 4 – Late primary spermatocytes; 5 – Round [file 12864_2020_7016_MOESM1_ESM.docx]

**Supplementary Figures and Methods**


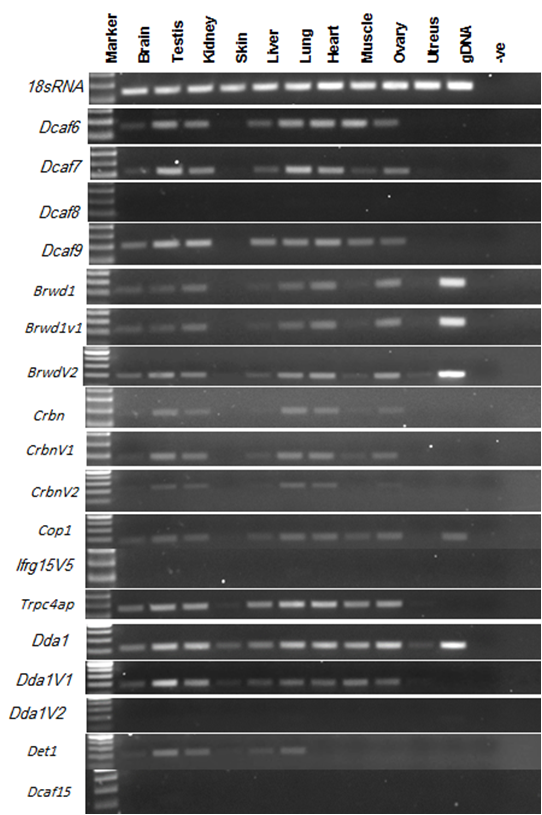


**A**

**Figure S1A: Agarose gel images of RT-PCR products of different DCAF genes from mouse (A).** RT-PCR was performed on total RNA from different tissues of adult C57BL/6 mouse using gene specific primers for 56 different DCAF subfamily genes as well as housekeeping genes (*β-actin* and *18S rRNA*). The PCR products were separated on 1.5% agarose gel and images were captured using gel imager. gDNA: Genomic DNA; -ve: No template control.


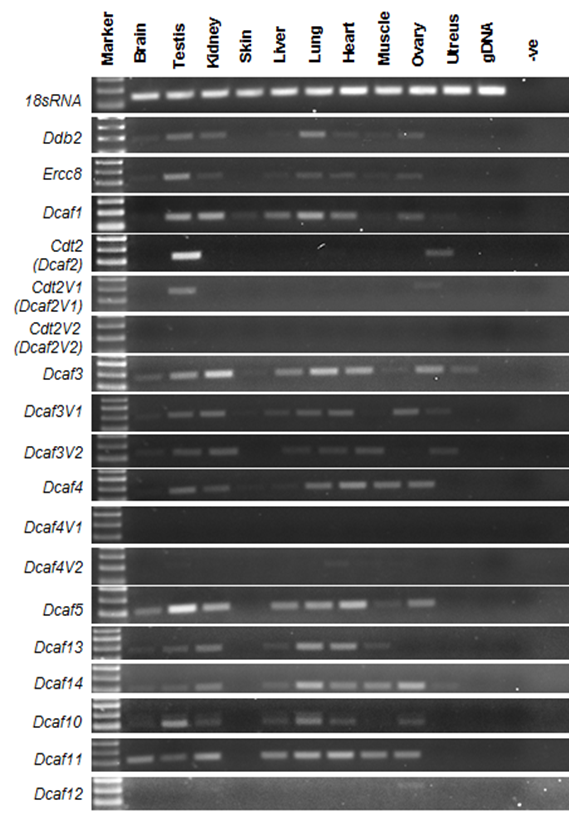

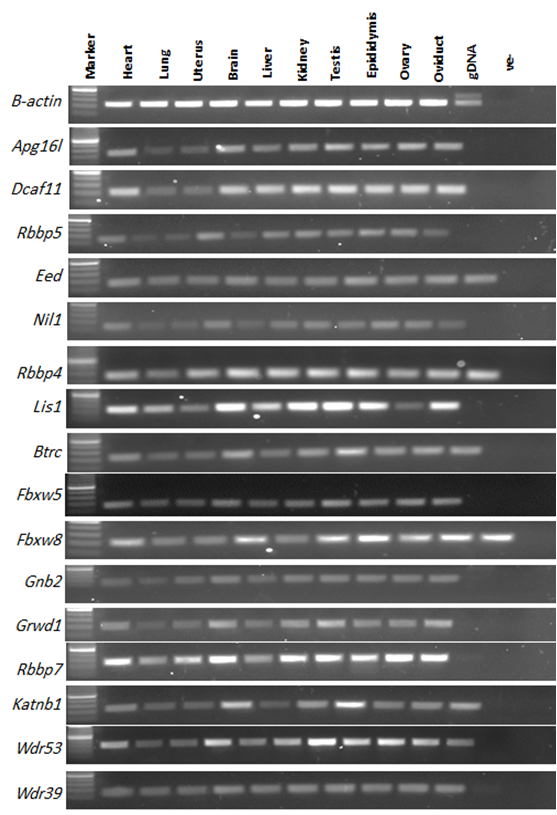

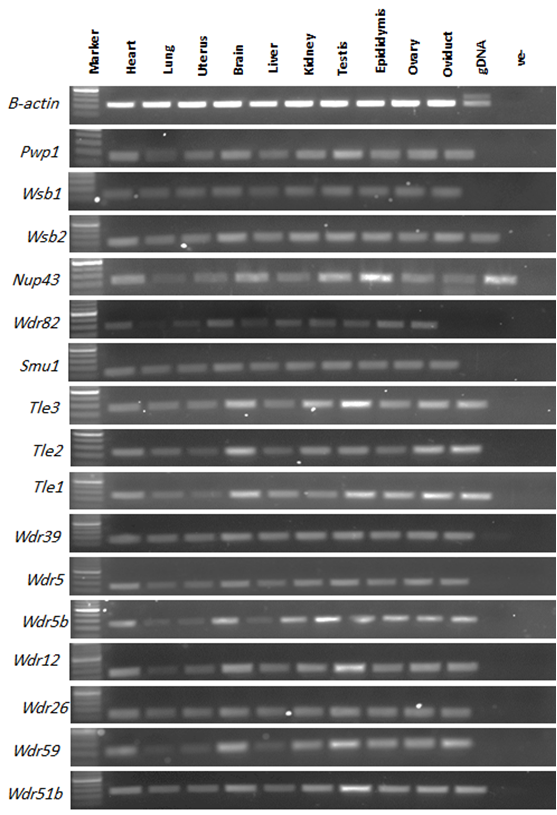

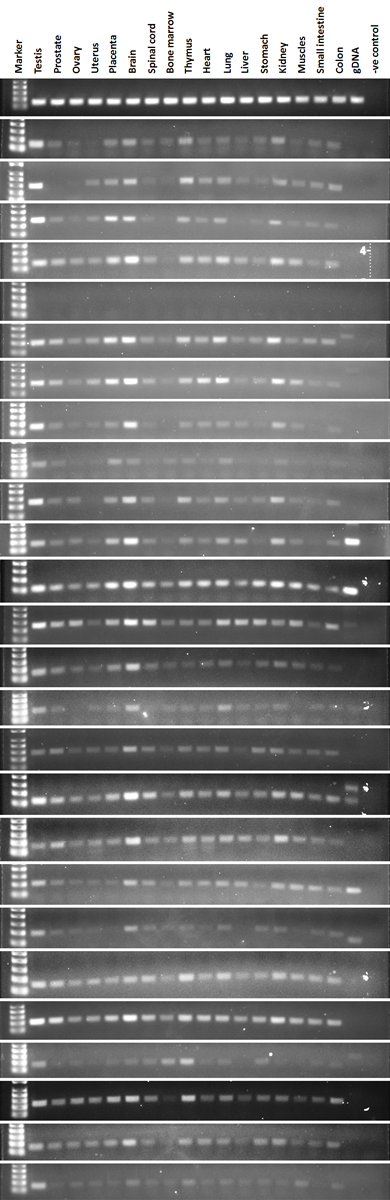

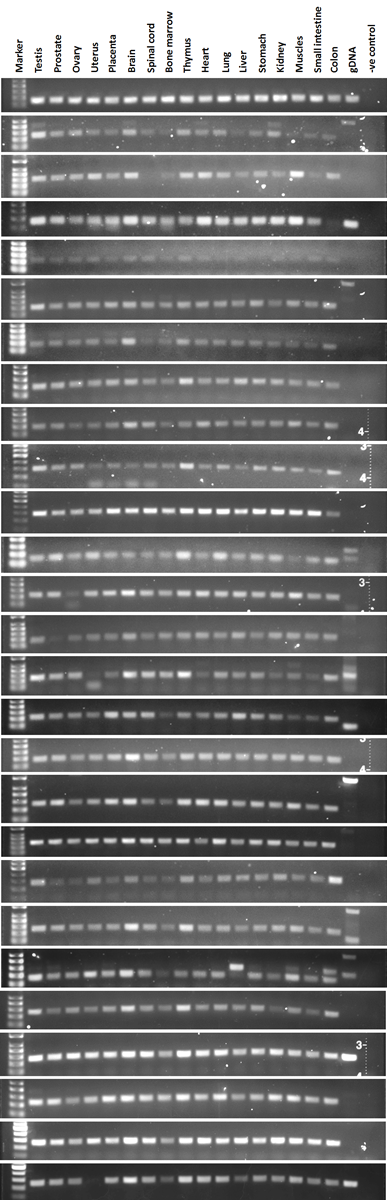


**Figure S1B: Agarose gel images of RT-PCR products of different *Dcaf* genes from human (B).** RT-PCR was performed on total RNA from different tissues of adult human using gene specific primers for 52 different DCAF subfamily genes as well as housekeeping genes (18S rRNA). The PCR products were separated on 1.5% agarose gel and images were captured using gel imager. gDNA: Genomic DNA; -ve control: No template control.

***18S rRNA***

***18S rRNA***

***ERCC8***

***COP1***

***DCAF1***

***DCAF5***

***DCAF6***

***DCAF7***

***DCAF9***

***DCAF10***

***DCAF12***

***DCAF13***

***DCAF19***

***DDA1***

***CRBN***

***BRTC***

***KATNB1***

***RBBP4***

***LIS1***

***WDR5***

***WDR12***

***WDR51B***

***WDR57***

***WDR61***

***WDR76***

***DCAF3***

***DCAF4***

***WDR53***

**B**

***DCAF16***

***DET1***

***FBXW5***

***FBXW8***

***RBBP7***

***NLE1***

***WDR59***

***WSB2***

***NUP43***

***GNB2***

***DDB2***

***DCAF8***

***DCAF11***

***DCAF14***

***IFRG15***

***TRPC4AP***

***GRWD1***

***WSB1***

***PWP1***

***RBBP5***

***TLE1***

***EED***

***WDR26***

***SMU1***

***WDR39***

***WDR82***


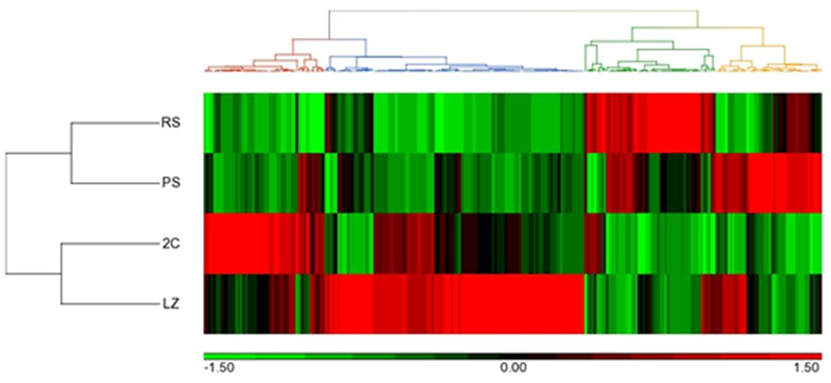


**Figure S2: Heat map of WD40 family genes (A) and DCAF subfamily genes (B) showing differential expression in different populations of purified spermatogenic cells from mouse testes.** Heat map of WD40 and DCAF genes generated from Cruz et al’s RNA sequencing dataset (da Cruz et al., 2016). Samples are denoted in rows and genes are denoted in columns. Red and green denote highly and weakly expressed genes, respectively. LZ - leptotene and zygotene; 2C - spermatogonia, secondary spermatocytes and Sertoli cells; PS - pachytene spermatocytes; RS - round spermatids.

**A**


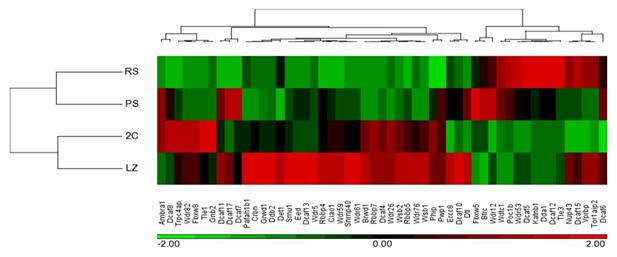


**B**


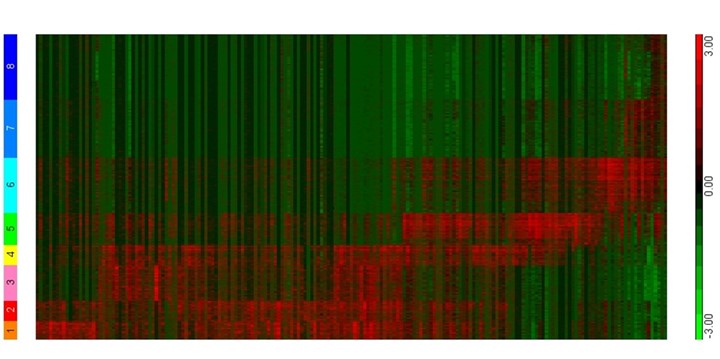

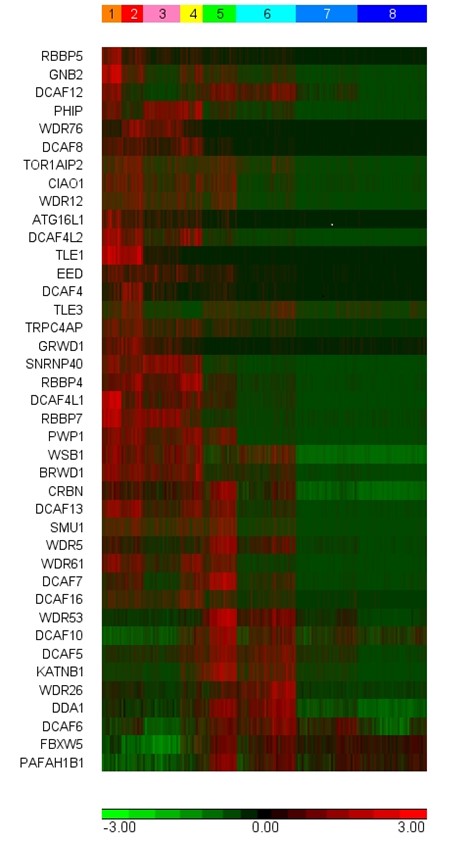


**Figure S3:** **Heat map of WD40 family genes (A) and DCAF subfamily genes (B) showing differential expression in different populations of purified spermatogenic cells from human testes.** Heat map of WD40 and DCAF genes generated from Guo et al’s RNA sequencing dataset (Guo et al., 2018). Samples are denoted in columns and genes are denoted in rows. Red and green denote highly and weakly expressed genes, respectively. 1 – Spermatogonial stem cells (SSCs); 2 – Differentiating spermatogonia; 3 – Early primary spermatocytes; 4 – Late primary spermatocytes; 5 – Round spermatids; 6 – Elongated spermatids; 7 & 8 – Sperm.

**A**

**B**


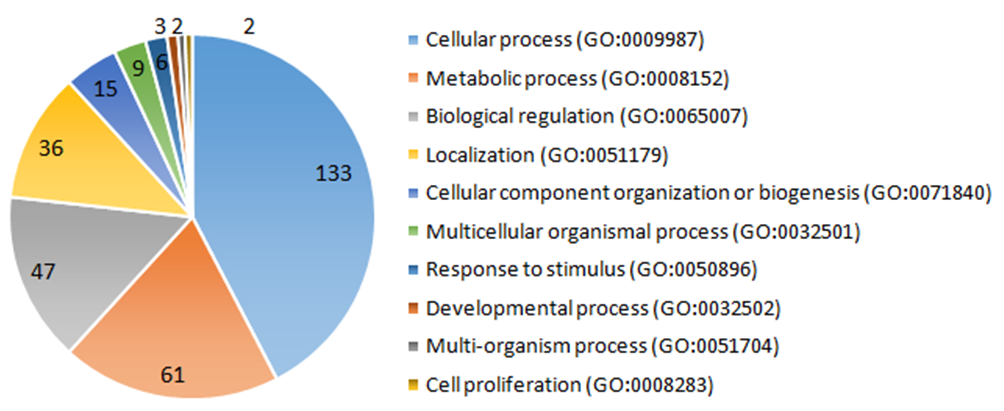


**Figure S4:  Pie charts showing the gene ontology (GO) categorization of the human WD40 family genes (A) and DCAF subfamily genes (B) according to biological processes.** WD40 and DCAF genes of human were classified into 10 GO categories for biological processes using Panther. Size of the pie slice corresponds to the number of genes in a given GO category.


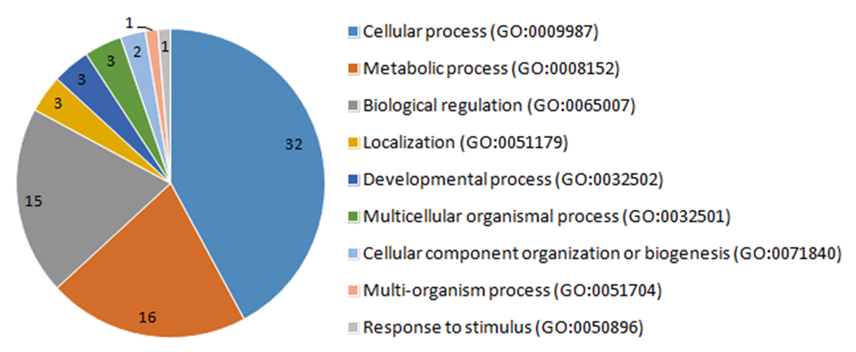


**A GO biological processes categorization of human WD40 genes**

**B GO biological processes categorization of human DCAF genes**


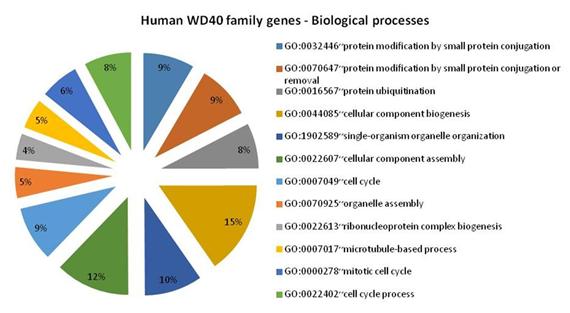

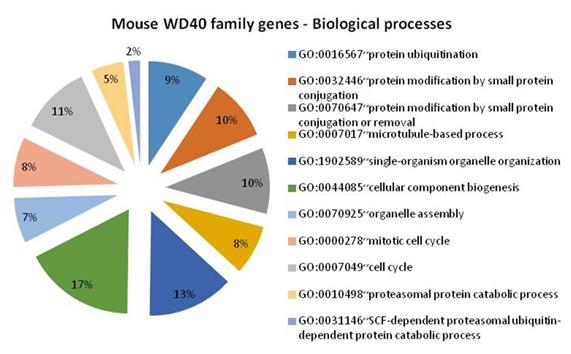

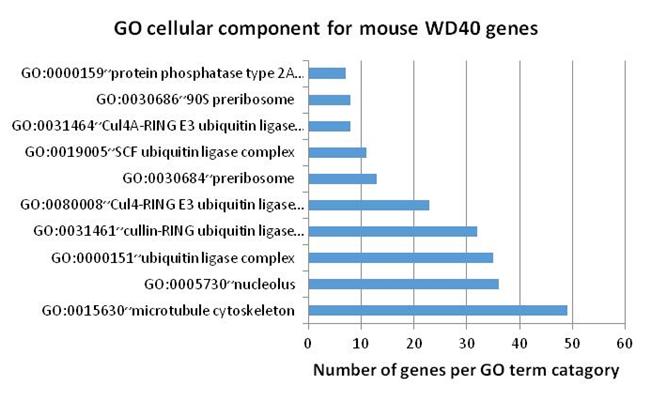

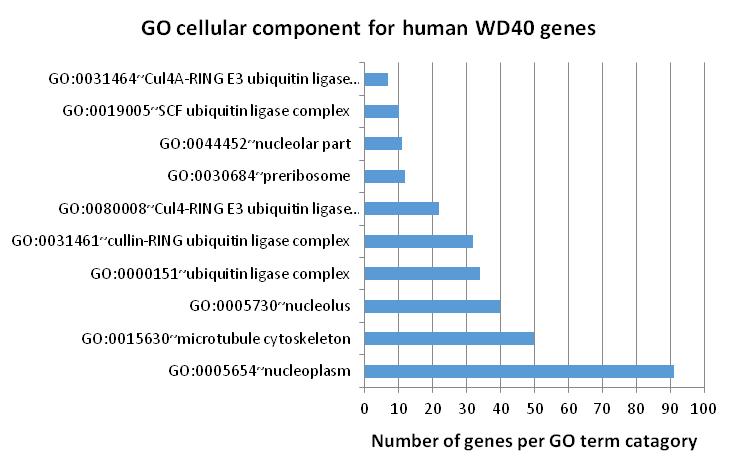

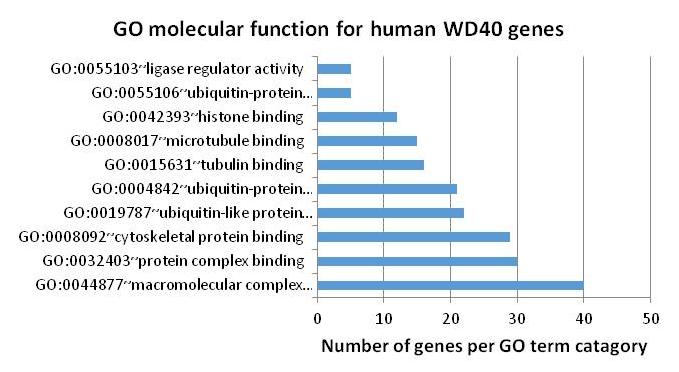

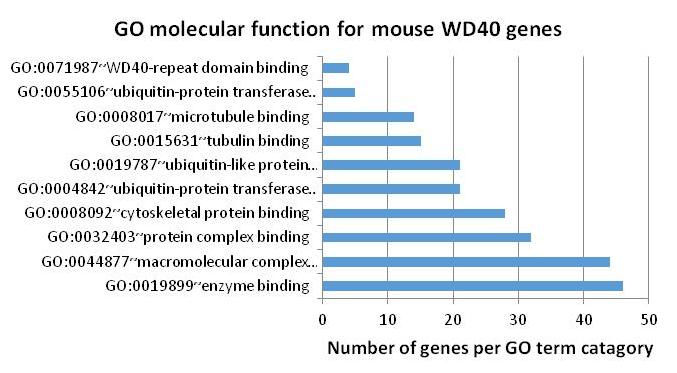


**Figure S5: Pie and bar charts representing the gene ontology (GO) categorization of the mouse(A, C, E) and human (B, D, F) WD40 family genes according to biological processes (A, B), cellular components (C, D) and molecular functions (E, F) using DAVID.**

**A**

**B**

**C**

**D**

**E**

**F**


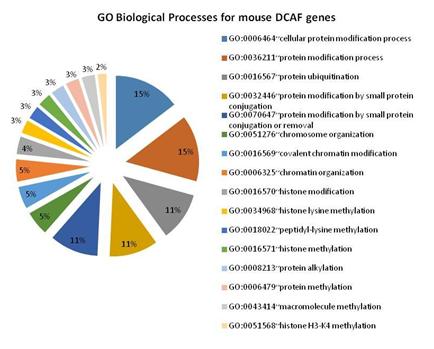

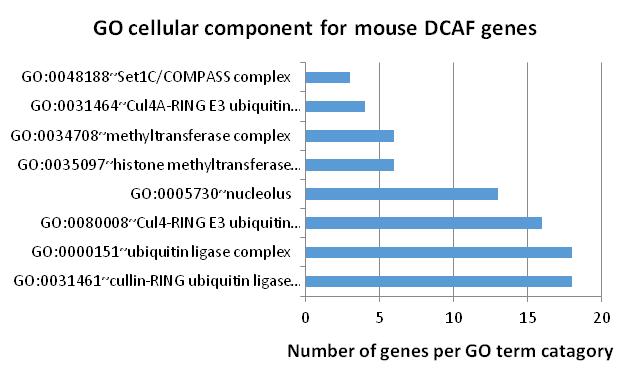

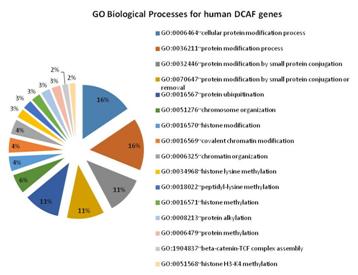

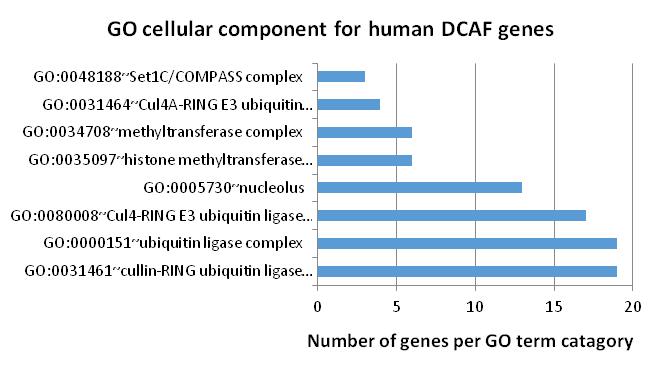

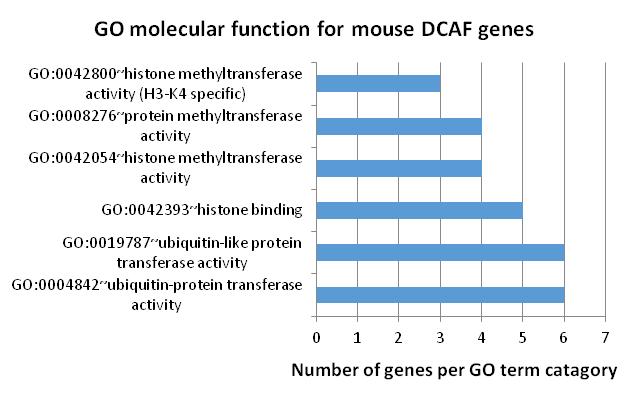

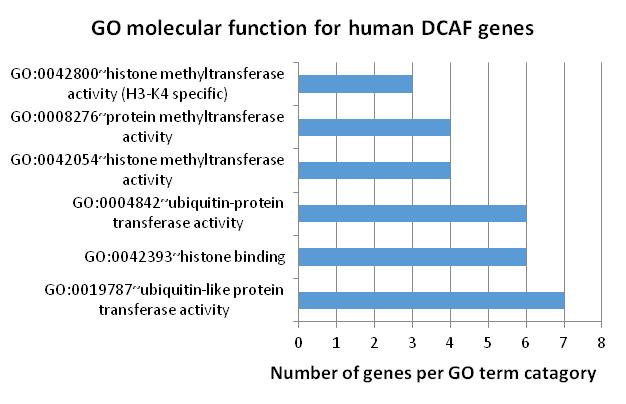


**Figure S6: Pie and bar charts representing the gene ontology (GO) categorization of the mouse (A, C, E) and human (B, D, F) DCAF family genes according to biological processes (A, B), cellular components (C, D) and molecular functions (E, F) using DAVID.**

**A**

**B**

**C**

**D**

**E**

**F**

**Methods**

**Semi quantitative reverse transcription-polymerase chain reaction (RT-PCR)**

The PCR amplifications were carried out in 25 µl reaction volumes containing 2.5 µl of 10Χ Qiagen PCR buffer (Tris·Cl, KCl, (NH4)_2_SO_4_, 15 mM MgCl_2_; pH 8.7), 0.5 µl dNTP mix (10 mM each), 2 µl gene specific forward and reverse primers (Table S7) mixture (5 µM each), 0.5 µl MgCl_2_ (25 mM), 3 µl cDNA (1:10 diluted) as a template, 16.3 µl nuclease-free water and 0.2 µl Taq DNA polymerase (25 Units). The PCR reaction mixtures were subjected to amplification cycles on BIO-RAD C1000 Touch™ thermal cycler (BIO-RAD, CA, USA) using the thermal cycler conditions of initial denaturation at 94°C for 15 min, 35 cycles of denaturation at 94°C for 30 sec, annealing at 59°C for 30 sec and extension at 72°C for 45 sec followed by final extension at 72°C for 10 min. The amplification products were subjected to agarose gel electrophoresis by loading 5 µl of PCR product mixed with 1 µl 6X loading dye (Promega) on 1% denaturing Tris-acetate-EDTA (TAE) agarose gel pre-stained with ethidium bromide. To estimate the size of DNA samples 100bp DNA ladder (Promega) was run on the agarose gel alongside the samples. To visualize PCR amplification product, the gel was exposed to UV light using an ImageQuant LAS 4000 (GE Life Sciences) imaging system and the gel images were captured using ImageQuant LAS 4000 software (GE Life Sciences).
